# Using RNA-Seq for gene identification, polymorphism detection and transcript profiling in two alfalfa genotypes with divergent cell wall composition in stems

**DOI:** 10.1186/1471-2164-12-199

**Published:** 2011-04-19

**Authors:** S Samuel Yang, Zheng Jin Tu, Foo Cheung, Wayne Wenzhong Xu, JoAnn FS Lamb, Hans-Joachim G Jung, Carroll P Vance, John W Gronwald

**Affiliations:** 1 USDA-Agricultural Research Service, Plant Science Research Unit, St. Paul, MN, 55108, USA; 2 Supercomputing Institute for Advanced Computational Research, University of Minnesota, Minneapolis, MN 55455, USA; 3 The J. Craig Venter Institute, Rockville, MD 20892, USA; 4 Department of Agronomy and Plant Genetics, University of Minnesota, St. Paul, MN 55108, USA; 5 Center for Human Immunology, Autoimmunity and Inflammation, National Institute of Health, Bethesda, MD 20892, USA

## Abstract

**Background:**

Alfalfa, [*Medicago sativa *(L.) sativa], a widely-grown perennial forage has potential for development as a cellulosic ethanol feedstock. However, the genomics of alfalfa, a non-model species, is still in its infancy. The recent advent of RNA-Seq, a massively parallel sequencing method for transcriptome analysis, provides an opportunity to expand the identification of alfalfa genes and polymorphisms, and conduct in-depth transcript profiling.

**Results:**

Cell walls in stems of alfalfa genotype 708 have higher cellulose and lower lignin concentrations compared to cell walls in stems of genotype 773. Using the Illumina GA-II platform, a total of 198,861,304 expression sequence tags (ESTs, 76 bp in length) were generated from cDNA libraries derived from elongating stem (ES) and post-elongation stem (PES) internodes of 708 and 773. In addition, 341,984 ESTs were generated from ES and PES internodes of genotype 773 using the GS FLX Titanium platform. The first alfalfa (*Medicago sativa*) gene index (MSGI 1.0) was assembled using the Sanger ESTs available from GenBank, the GS FLX Titanium EST sequences, and the *de novo *assembled Illumina sequences. MSGI 1.0 contains 124,025 unique sequences including 22,729 tentative consensus sequences (TCs), 22,315 singletons and 78,981 pseudo-singletons. We identified a total of 1,294 simple sequence repeats (SSR) among the sequences in MSGI 1.0. In addition, a total of 10,826 single nucleotide polymorphisms (SNPs) were predicted between the two genotypes. Out of 55 SNPs randomly selected for experimental validation, 47 (85%) were polymorphic between the two genotypes. We also identified numerous allelic variations within each genotype. Digital gene expression analysis identified numerous candidate genes that may play a role in stem development as well as candidate genes that may contribute to the differences in cell wall composition in stems of the two genotypes.

**Conclusions:**

Our results demonstrate that RNA-Seq can be successfully used for gene identification, polymorphism detection and transcript profiling in alfalfa, a non-model, allogamous, autotetraploid species. The alfalfa gene index assembled in this study, and the SNPs, SSRs and candidate genes identified can be used to improve alfalfa as a forage crop and cellulosic feedstock.

## Background

The advent of next generation high-throughput sequencing has revolutionized the analysis of genomes and transcriptomes [1-5]. When applied to the transcriptome, this methodology is referred to as RNA-Seq (RNA sequencing). RNA-Seq has been used for gene annotation, expression analysis and SNP discovery [6,7]. This methodology has also proven useful for discovery of novel transcripts (coding and non-coding) and identification of alternative splice variants [5,8]. It is expected that RNA-Seq methodologies will supersede microarrays for transcript profiling because of higher sensitivity, base-pair resolution and the larger range of expression values that can be detected [3,5,9]. Furthermore, in contrast to microarrays, RNA-Seq does not require prior knowledge of gene sequences. However, RNA-Seq presents bioinformatic challenges because of the required assembly of millions of short sequence reads that are generated by the methodology.

RNA-Seq has been successfully used for annotation, transcript profiling and/or SNP discovery in a number of plant species. For model plant species with sequenced genomes, sequence reads can be mapped to the reference genome. The model species where RNA-Seq analysis has been applied include *Arabidopsis *[10,11], soybean [12,13], rice [14], maize [15] and *Medicago truncatula *[16]. There are also examples of the application of RNA-Seq to non-model plant species that lack a reference genome. In the absence of a reference genome, *de novo *assembly of sequence reads into contigs is required. RNA-Seq has been used for transcript profiling in *Eucalyptus grandis *[17], grape (*Vitis vinifera *L.) [18], California poppy (*Eschschlozia califonica*) [11], avocado (*Persea americana*) [11], *Pachycladon enysii *[19] and *Artemisia annua *[20]. In *Eucalyptus grandis *and rape (*Brassica napus*), RNA-Seq was used for SNP discovery [17,21].

Alfalfa is the most widely cultivated forage legume in the world and the fourth most widely grown crop in the US [22,23]. In addition to its value as a livestock feed, alfalfa also has potential as a cellulosic ethanol feedstock [24,25]. Alfalfa is an allogamous autotetraploid with complex polysomic inheritance [26-28]. Slow progress has been made in improving the agronomic traits of this species using traditional breeding approaches based on phenotypic selection. For the most part, genomic approaches for crop improvement (e.g., molecular breeding) have not been applied to this legume because of limited genomic resources. As of February 2010, there were 12,371 alfalfa ESTs available in the public database. A few SSRs have been detected but SNPs have not yet been identified [28-30]. Recently, we reported on the results of transcript profiling and single feature polymorphism (SFP) detection in alfalfa using the *Medicago *GeneChip as a cross-species platform [25,31]. The *Medicago *GeneChip contains probe sets designed for the model plant, *Medicago truncatula*, a diploid relative of alfalfa. Using a method based on probe affinity differences and affinity shape power, we identified over 10,000s SFPs in the stem internodes of alfalfa genotypes 252 and 1283 that differed in cellulose and lignin concentrations in cell walls [31]. In a subsequent study using the *Medicago *GeneChip for transcript profiling of alfalfa genotypes 252 and 1283, interspecies variable regions and SFPs were masked prior to data analysis resulting in a 2-fold increase in the number of differentially expressed genes detected in stem internodes of the two genotypes [25]. Although the research of Yang et al. [25,31] significantly advanced alfalfa genomics, the use of a cross-species platform for microarray analysis limits the sensitivity and specificity of transcriptome analysis and polymorphism detection.

The stem tissue of alfalfa is important in determining the value of this forage as a livestock feed and cellulosic feedstock. Increasing the cellulose and decreasing the lignin content in cell walls in stems would improve alfalfa for both uses. In this study, we applied RNA-Seq to gene identification, polymorphism detection and transcript profiling of two alfalfa clonal lines (708, 773) that differ in cell wall composition in stems. The results were used to assemble the first gene atlas for alfalfa (MSGI 1.0). Our research also provides the first report of high-throughput SNP detection and digital gene expression analysis in the alfalfa transcriptome.

## Results and discussion

### Cell wall composition of stems of genotypes 708 and 773

**Figure 1:**
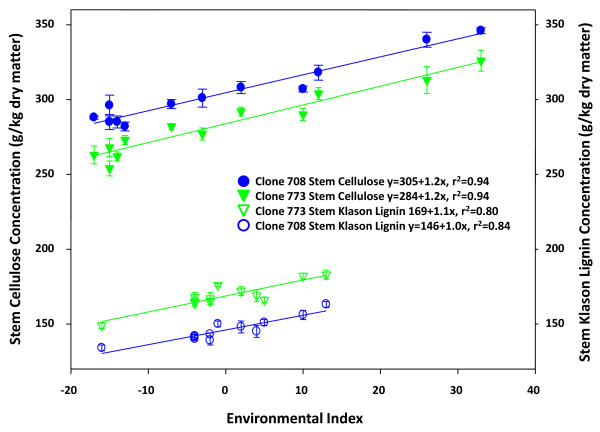
**Regression analyses of cellulose and Klason lignin concentrations in stems of two alfalfa genotypes**. The stems of genotype 708 were consistently higher in cellulose and lower in Klason lignin compared to stems of genotype 773 across twelve environmental indexes (field environments). The high r^2 ^values for all regression lines suggest that genotypic differences in stem cellulose and Klason lignin concentrations were environmentally stable.

The alfalfa genotypes 708 and 773 used in this study were selected for divergent cell wall composition in stems under field conditions (see Methods for details). Cell wall composition of greenhouse grown stems used for RNA sampling in the current study is shown in Table 1. Cell wall concentration in stems of the two clones did not differ. In contrast, cellulose content (defined as glucose) in the stems of genotype 708 was 5.2% greater compared to genotype 773 (*p *< 0.05) (Table 1). In addition, galactose and mannose concentrations were 14.2% (*p *< 0.05) and 8.5% (*p *< 0.01) greater, respectively, in stems of genotype 708 compared to genotype 773 (Table 1). Klason lignin concentration in the cell wall was 8.0% greater in stems of 773 compared to stems of 708 (*p *< 0.05) (Table 1). These genotypes consistently displayed differences in cell wall cellulose and lignin content in stems when plants were grown under different field environments (Figure 1) and in the greenhouse (Table 1).

**Table 1:** Comparison of cell wall components in stems of genotypes 708 and 773 on a cell wall basis.

Component	Genotype 708	Genotype 773	SEM	*p*-value
	---------- g kg^-1 ^cell wall ----------			
Klason lignin	162	175	2	*p *< 0.05
Glucose	443	421	2	*p *< 0.05
Xylose	137	149	3	NS
Arabinose	39	39	1	NS
Galactose	32	28	1	*p *< 0.05
Mannose	33.1	30.5	0.1	*p *< 0.01
Rhamnose	11.5	11.4	0.4	NS
Fucose	3.01	3.1	0.03	NS
Uronic acids	139	142	6	NS

Values are least square means based on an analysis of variance with three biological replicates for each clone arranged in a randomized complete block design (see Methods for details). SEM = Standard error of mean, NS = Non-significant (*p *> 0.05).

### RNA-Seq using the Illumina GA-II platform

For RNA-Seq analysis, we developed a total of four cDNA libraries derived from elongating stem (ES) and post-elongation stem (PES) internodes of alfalfa genotypes 708 and 773 (see Methods for details). In alfalfa stems, genes associated with primary cell wall development are preferentially expressed in ES internodes while genes associated with secondary xylem development are enriched in PES internodes [25]. For sequencing by synthesis using the Illumina GA-II platform, cDNA libraries 708ES, 708PES and 773ES were run on two lanes per library while the 773PES library was run on one lane. A total of 234,908,899 EST reads were generated by a single run of 76 cycles. After filtering low quality reads, a total of 198,861,304 reads (76-bp in size) were selected for further analysis (see Methods for details). The Illumina reads generated in this study are available at the NCBI SRA browser (accession number GSE26757; http://www.ncbi.nlm.nih.gov/geo/query/acc.cgi?acc=GSE26757.

*de novo *assembly of short RNA-Seq reads without a known reference is a challenging task especially for alfalfa, an allogamous autotetraploid with complex polysomic inheritance. In this study, we used the Velvet algorithm [32] for *de novo *assembly of the 198,861,304 Illumina reads (76 bp) into a total of 132,153 unique sequences with an average length of 284 bp (Additional file 1). The Velvet algorithm has also been used successfully for *de novo *transcriptome assembly in previous studies [33,34]. The Velvet algorithm was originally developed for *de novo *assembly of genome sequences where the coverage is expected to be homogeneous throughout the genome. However, the coverage of transcripts is highly heterogeneous due to difference in gene expression. Previous studies showed that *de novo *assembly using the Velvet program with longer k-mers results in a more contiguous transcript assembly but lower transcript diversity compared to shorter k-mers [32,33]. Although several recent studies introduced new algorithms and methodologies developed for *de novo *transcriptome assembly [35-38], a consensus standard protocol has not yet emerged for *de novo *transcriptome assembly. In this study, we optimized our Velvet *de novo *transcriptome assembly to favor transcript contiguity with high specificity as opposed to increased transcript diversity (see Methods for details). To complement the limitation of the high k-mer that we selected for the Velvet assembly in this study (lower diversity and probably biased toward highly expressed genes), we generated additional ESTs using the GS FLX Titanium platform.

### RNA-Seq using the GS FLX Titanium platform

We generated a total of 341,984 additional ESTs (average length 243 bp, minimum length 40 bp, maximum length 792 bp) using the GS FLX Titanium platform http://www.454.com. The additional EST sequences were generated from the cDNA libraries derived from ES (124,533 ESTs, average length 230 bp) and PES (217,451 ESTs, average length 256 bp) internodes of the genotype 773. The additional ESTs obtained using the GS FLX Titanium platform increased the diversity of transcripts discovered and hence provided broader coverage of the alfalfa transcriptome than would have been achieved based on the *de novo *assembly of the Illumina reads alone. The additional ESTs are also available at the NCBI SRA browser (accession number GSE26757; http://www.ncbi.nlm.nih.gov/geo/query/acc.cgi?acc=GSE26757.

### Alfalfa Gene Index 1.0 (MSGI 1.0)

We used the Gene Index Assembly protocol [39,40] for reference transcriptome assembly in alfalfa. This protocol has been used for over a decade to build unigene assemblies for numerous species of animals, plants and microorganisms http://compbio.dfci.harvard.edu/tgi/plant.html. However, no gene index is currently available for alfalfa. In this study, the first alfalfa (*Medicago sativa*) gene index (MSGI 1.0) was built by combining the *de novo *assembled Illumina reads using the Velvet program (132,153 sequences), the 341,984 ESTs obtained using the GS FLX Titanium platform, and 12,371 Sanger ESTs for alfalfa available in the public database http://www.ncbi.nlm.nih.gov following the Gene Index Assembly protocol previously described [39,40].

MSGI 1.0 contains a total of 124,025 unique sequences including 22,729 tentative consensus sequences (TCs), 22,315 singletons and 78,981 pseudo-singletons (Additional file 2). Pseudo-singletons refer to the *de novo *assembled Illumina sequences that were not assembled into contigs during the Gene Index Assembly process. The average length of the unique sequences in MSGI 1.0 is 384 bp. Unique sequence lengths ranged from 100 to 6,956 bp with more than 10,000 sequences larger than 800 bp. The total base count of the sequences in MSGI 1.0 is 47,628,953 bp. The newly built alfalfa gene index increases the number of alfalfa sequences publicly available by about 10-fold.

### Gene annotation and functional classification

**Figure 2:**
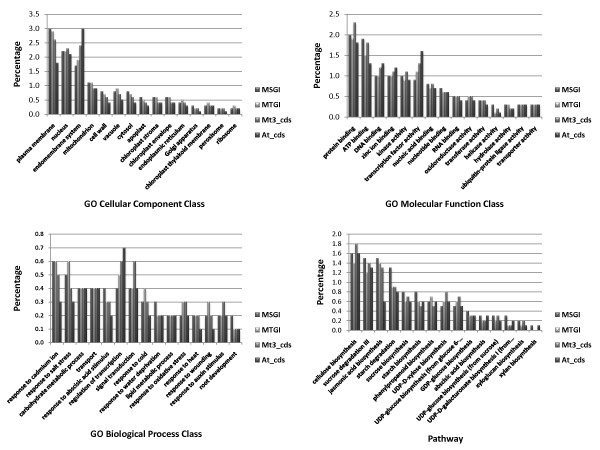
**Comparison of percentage distribution of gene ontology and pathway classifications using four reference databases**. The percentage distributions of gene ontology (GO) classes and pathways are shown for the following reference databases: (1) the *Medicago sativa *Gene Index (MSGI 1.0) assembled in this study, (2) the *Medicago truncatula *Gene Index (MTGI 9.0), (3) the *M. truncatula *coding sequences (Mt3.0 cds), and (4) the *Arabidopsis *coding sequences (At cds).

We assigned putative functions for the unique sequences in MSGI 1.0 by conducting BlastX searches against the non-redundant (NR) protein database (e-value cutoff of 1e-10) (Additional file 3). Putative functions could be assigned for about 83% of the sequences. We also assigned gene ontology (GO) functional classes and MapMan functional classifications [41] to the unique sequences in MSGI 1.0 (Additional file 3) (see Methods for details). To examine whether bias occurs among the functional classes represented in MSGI 1.0, we compared the percentages of each GO functional class and pathway in MSGI 1.0 with the percentages found in the *M. (Medicago) truncatula *Gene Index (MTGI 9.0), the *M. truncatula *coding sequences (Mt3.0 cds) and the *Arabidopsis *coding sequences (At cds) (Figure 2). Although most of the sequences in MSGI 1.0 were derived from stem tissues, similar levels of representation of most functional classes were found in MSGI 1.0 and the other databases (MTGI 9.0, Mt3.0 cds, and At cds). These results suggest that MSGI 1.0 can serve as a reference sequence database for genomic analysis in alfalfa.

### SSR detection

We detected simple sequence repeats (SSRs) among sequences in MSGI 1.0 using the MISA program [42] (see Methods for details). A total of 1,294 SSRs were identified among 1,245 sequences which represents about 1.7% of the total unique sequences in MSGI 1.0 (Additional file 4). The estimated frequency of SSRs among the expressed sequences was one SSR per 37 kb. SSR detection frequency is dependent on the SSR detection parameter [43]. The SSR frequency measured in this study is significantly lower than that detected in other species (one SSR per 11 kb) where the same SSR detection parameter was used [40]. The significantly reduced SSR detection frequency found in MSGI 1.0 sequences may be due to the reduced detection efficiency of short length sequences (384 bp on average for MSGI1.0). Alternatively, the SSR frequency among expressed sequences may be lower in alfalfa compared to other species. SSRs with mono-, di-, tri-, tetra-, penta- and hexanucleotide repeats composed about 5.4%, 30.4%, 47.2%, 10.6%, 3.9% and 2.5% of the SSRs in MSGI 1.0, respectively. Using the default parameter of the Primer3 program [44], we designed SSR primers spanning a total of 664 SSRs (Additional file 4).

### SNP detection

To identify SNPs between alfalfa genotypes 708 and 773, Illumina EST reads from ES and PES internode libraries were combined for each genotype. The combined ES and PES reads for each genotype were independently aligned to the MSGI 1.0 sequences using the Maq program [45]. From the alignment output of each genotype, we summarized the depth (frequency) of each nucleotide (A, G, C, or T) at each base position in each reference sequence. Next, to reduce the identification of false positive SNPs, we filtered potential SNPs using a stringent nucleotide depth cutoff of 10 [e.g., at least 10 adenines (A) in one genotype vs. at least 10 guanines (G) in the other genotype] for each genotype (see Methods for details). Using this protocol, we identified 10,826 SNPs between genotypes 708 and 773 in 7,282 sequences in MSGI 1.0 (Additional file 5). About 74% of these sequences contained a single SNP while about 2.3% contained 5 or more SNPs.

To validate the SNPs that were predicted using the RNA-Seq data generated in this study, we randomly selected 55 SNPs. Genomic DNAs purified from genotypes 708 and 773 were genotyped by MALDI-TOF mass spectrometry using the iPLEX Gold spectrometry system http://www.sequenom.com. Out of 55 SNPs tested, 47 (85%) were polymorphic between the two genotypes (Additional file 6). In addition to genotypes 708 and 773, we also genotyped 51 additional alfalfa (*M. sativa*) genotypes selected from different populations of *M. sativa ssp. sativa *or *M. sativa ssp. falcata*. The 47 validated SNPs between 708 and 773 also showed polymorphism among the other *Medicago *genotypes tested (Additional file 6). This suggests that the SNPs predicted in this study can also be used for genotyping in other alfalfa genotypes.

**Figure 3:**
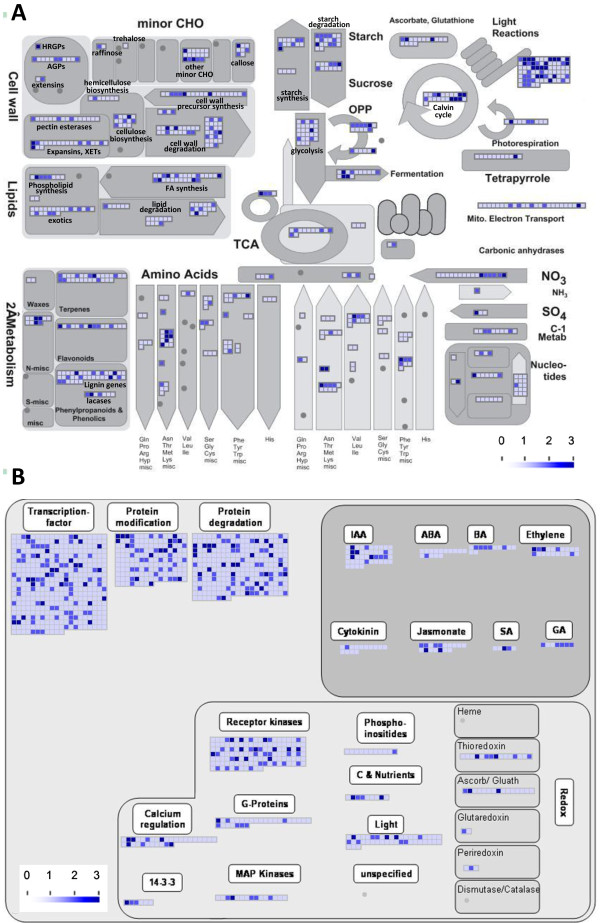
**MapMan overview of cellular metabolism (A) and regulation (B) showing SNP-harboring genes and SNP frequencies**. Individual genes are represented by small squares. The SNP frequency for each gene is indicated by the intensity of the blue color on a 0 to 3 scale. Dark blue (scale intensity 3) indicates genes with three or more SNPs. A complete list of SNP-harboring genes, corresponding MapMan functional categories and SNP frequencies are provided in Additional file 5.

In a previous study that described single-feature polymorphism (SFP) discovery in alfalfa using the *Medicago *GeneChip as a cross-species platform [31], we proposed candidate gene-based association mapping for selecting alfalfa germplasm with modified cell wall composition in stems. In this study, SNPs were also identified in genes with various functional classes including numerous cell wall-related genes (Figure 3A). For example, SNPs were identified in 14 genes involved in cellulose biosynthesis including 11 cellulose synthase and three *COBRA *genes [46] (Figure 3A). In addition, SNPs were identified in 21 lignin pathway genes, 20 genes involved in cell wall precursor pathways (Figure 3A) and in numerous regulatory genes including various transcription factor families, signalling genes and hormone genes (Figure 3B).

To detect functional classes over- or under-represented among the SNP-harboring genes, we performed Fisher's exact test with Bonferroni correction (z-value cutoff = 1) as previously described [31] (Additional file 7). The functional classes over-represented among SNP-harboring genes included photosynthesis, cell wall, amino acid metabolism, stress response (biotic and abiotic), nodulin-like, protein synthesis and WRKY transcription factor classes (Additional file 7). The SNPs developed in this study can be used for either candidate gene-based or whole genome scanning association mapping studies to identify SNPs associated with cell wall traits in alfalfa stems. With further development, the SNPs identified in this study may prove to be useful in molecular breeding programs focused on improving alfalfa as a forage crop and biomass feedstock via marker-assisted selection.

In this study, we also identified allelic variations (SNPs) within genotypes. Using a minimum SNP depth cutoff of 10, we detected 287,555 and 168,966 allelic variations (SNPs) within genotypes 708 and 773, respectively (Additional files 8 and 9). These SNPs within genotype were detected in 55,320 and 33,406 sequences for genotypes 708 and 773, respectively. Detection of allelic variations (SNPs) within genotypes is equally important as detecting SNPs between genotypes for understanding phenotypic differences (e.g. cell wall composition) and for future applications such as marker-assisted selection.

### Comparison of MSGI 1.0 and Mt3.0 cds as reference sequences for digital transcript profiling

The alfalfa gene index (MSGI 1.0) developed in this study provides a reference sequence database that can be used for digital gene expression analysis in alfalfa. However, another option for RNA-Seq analysis in alfalfa is to use Mt3.0 cds as a reference sequence because *M. truncatula and *alfalfa share significant coding sequence homology [25]. Furthermore, sequences in Mt3.0 cds are full-length sequences (predicted gene models) with better coverage than sequences in MSGI 1.0 where the majority are partial sequences. As an initial step to evaluate the utility of MSGI 1.0 and Mt3.0 cds as reference sequences for transcript profiling of alfalfa, the Illumina EST reads generated in this study were mapped to MSGI 1.0 and Mt3.0 cds sequences using the bowtie program [47] (see Methods for details). On average, about 70% of the EST reads in each library (708 ES, 773 ES, 708 PES, and 773 PES) could be mapped to the MSGI 1.0 sequences. In contrast, only about 30% of the EST reads could be mapped to the Mt3.0 cds sequences (data not shown). We measured the raw digital expression counts for each gene by quantifying the number of EST reads that were mapped to each reference sequence. The raw digital gene expression counts were normalized using the RPKM (reads/Kb/Million) method [1,48] to correct the digital gene expression counts for bias caused by reference sequence size and total EST numbers per library (see Methods for details).

**Figure 4:**
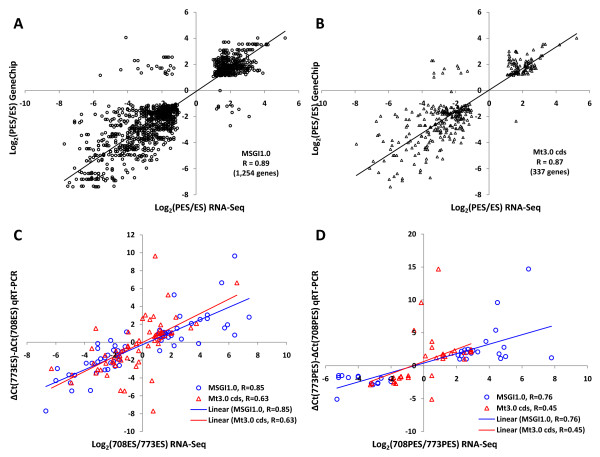
**Comparison of MSGI 1.0 and Mt3.0 cds as reference sequences for digital gene expression analysis**. For a subset of genes involved in stem development independent of genotypic variation, Log_2_(PES/ES) values from the RNA-Seq data (*x-axis*) generated using (A) the *Medicago sativa *Gene Index (MSGI1.0) or (B) the *Medicago truncatula *coding sequences (Mt3.0 cds) as reference sequences were plotted against Log_2_(PES/ES) values from the GeneChip data (*y-axis*) previously generated [25]. For 63 randomly selected genes (C) and 34 selected cell wall genes (D), Log ratio values from the RNA-Seq data (*x-axis*) generated using MSGI1.0 (**O**) and Mt3.0 cds (**Δ**) as reference sequences were plotted against ΔΔC_T _values obtained from the qRT-PCR data (*y-axis*).

Further evaluation of MSGI 1.0 and Mt3.0 cds as reference sequence databases for alfalfa was conducted by comparing RNA-Seq data with the previously generated GeneChip data for the same stem tissues but in different alfalfa genotypes [25] (see Methods for details). The RNA-Seq data generated using MSGI 1.0 or Mt3.0 cds showed a linear relationship with GeneChip data with similar Pearson correlation coefficients (R = 0.89 and R = 0.87, respectively) (Figure 4A and 4B). A total of 1,254 genes were commonly-selected between RNA-Seq and GeneChip data when MSGI 1.0 was used as reference sequences (Figure 4A). However, when Mt3.0 cds was used as reference sequences, the number of genes commonly-selected between RNA-Seq and GeneChip data decreased to 337 reflecting a significant decrease in detection sensitivity (Figure 4B). This is not surprising because, as described above, only about 30% of the EST reads could be mapped to the Mt3.0 cds while about 70% of the EST reads could be mapped to the MSGI 1.0 (data not shown).

As a final evaluation of MSGI 1.0 and Mt3.0 cds as reference sequences for digital gene expression analysis in alfalfa, we compared the digital gene expression data generated using MSGI 1.0 and Mt3.0 cds sequences with real -time quantitative RT-PCR (qRT-PCR) data obtained from 97 genes (63 randomly selected, 34 cell wall genes) (Additional file 10) (see Methods for details). Previous studies showed a linear relationship between ΔΔC_T _values from qRT-PCR and the log gene expression ratio obtained in microarray analysis [25,49,50]. We plotted ΔΔC_T _values obtained from the qRT-PCR data for randomly selected genes against Log_2_(708ES/773ES) values from the RNA-Seq data with MSGI 1.0 or Mt3.0 cds as reference sequences. The results showed a linear relationship between qRT-PCR data and the RNA-Seq data using both reference sequences. However, using MSGI 1.0 increased the Pearson correlation coefficient (R) from 0.63 to 0.85 (Figure 4C). Next, we plotted ΔΔC_T _values obtained from the qRT-PCR data for selected cell wall genes against Log_2_(708PES/773PES) values from the RNA-Seq data. Using MSGI 1.0 as the reference sequence database also increased the Pearson correlation coefficient (R) for selected cell wall genes from 0.45 to 0.76 (Figure 4D). On the basis of these results, we chose to use MSGI 1.0 as reference sequences for digital gene expression analysis of stems of alfalfa genotypes 708 and 773.

### Transcript profiling of stems of alfalfa genotypes 708 and 773

For transcript profiling of stems of alfalfa genotypes 708 and 773, we analyzed the RPKM-normalized digital gene expression counts for each sequence in MSGI 1.0 for cDNA libraries derived from ES and PES internodes of each genotype (Additional file 11). Among the 124,025 sequences in MSGI 1.0, about 94.7% were transcriptionally active (RPKM > 0) in at least one library while about 5.3% (6,629 sequences) were silent in all four libraries examined (RPKM = 0 in all 4 libraries) (Additional file 11).

Among the transcriptionally-active genes in each library, we identified the top 500 most abundant transcripts (Additional file 12). The Fisher's exact test with Bonferroni correction (z-value cutoff = 1) revealed that genes belonging to photosynthesis, amino acid metabolism and transport classes were significantly over-represented among the most abundantly expressed transcripts in all 4 libraries which suggests roles as housekeeping genes in alfalfa stems (Additional file 13). We also identified functional classes over-represented among the most abundant genes expressed in a genotype- or tissue-specific manner suggesting their role in determining genotype or tissue identity (Additional file 13). Interestingly, genes involved in lignin biosynthesis were significantly over-represented among the most abundant genes. The lignin genes over-represented in one or more libraries include CCoAOMT (caffeoyl-CoA O-methyltransferase), CCR1 (cinnamoyl-CoA reductase1) and COMT (caffeic acid O-methyltransferase) genes (Additional file 13). On the other hand, the transcription factor family class was significantly under-represented among the most abundant transcripts in three libraries (Additional file 13). Table 2 shows the top 10 most abundant protein-coding transcripts identified in each alfalfa stem internode library. Interestingly, a putative COMT gene (MSGI1_1270) was among the top 10 most abundant protein-coding transcripts and it was up-regulated in 773 (high lignin genotype) in both ES and PES internodes compared to 708 (low lignin genotype). The promoters of these highly expressed genes, including strong constitutive and tissue-specific promoters, may be useful for transgenic studies in alfalfa.

**Table 2:** Top 10 most abundant protein-coding transcripts identified in each alfalfa stem internodes library.

Unique_ID	Libraries				Putative Functions
		
	708_ES	708_PES	773_ES	773_PES	
------------ RPKM-normalized expression counts ------------					
MSGI1_2417	**6068 (1)**^†^	**6754 (1)**	**3271 (2)**	**6034 (1)**	Leucine-rich repeat family protein
MSGI1_8746	**4812 (2)**	**4697 (3)**	**3850 (1)**	**3586 (2)**	Chlorophyll a/b binding protein
MSGI1_523	**4213 (3)**	**5428 (2)**	**2719 (6)**	**3552 (3)**	Beta ketoacyl CoA synthase
MSGI1_18145	**3859 (4)**	979	**2555 (8)**	**3400 (4)**	Rubisco small chain
MSGI1_27309	748	**2453 (7)**	1317	**3336 (5)**	Metallothionein
MSGI1_11989	**2315 (6)**	**2574 (5)**	1171	**2387 (6)**	Uncharacterized protein
MSGI1_6529	265	328	**2393 (9)**	**2350 (7)**	Glycine rich protein
MSGI1_1166	1458	1486	901	**2182 (8)**	AAA ATPase
MSGI1_62398	160	225	1267	**2168 (9)**	Stress (ABA)-inducible protein
MSGI1_21335	**2012 (8)**	**2465 (6)**	1275	**2155 (10)**	Cytochrome P450-like
MSGI1_8707	**2762 (5)**	**2632 (4)**	1854	1833	Chlorophyll a/b binding protein
MSGI1_4749	1425	**1682 (10)**	1298	1693	Polyubiquitin
MSGI1_5229	**2287 (7)**	**2140 (8)**	1507	1561	Chlorophyll a/b binding protein
MSGI1_1270	744	969	**3145 (3)**	1470	Caffeic acid O-methyltransferase
MSGI1_1415	**1723 (9)**	1215	764	1468	Elongation factor 1-alpha
MSGI1_36219	1633	**1777 (9)**	723	1256	Uncharacterized protein
MSGI1_5153	**1705 (10)**	1531	1058	1090	Chlorophyll a/b binding protein
MSGI1_29285	324	513	**2861 (4)**	1035	Stress (ABA)-inducible protein
MSGI1_13276	86	77	**2750 (5)**	423	Cold acclimation responsive protein
MSGI1_7576	274	114	**2357 (10)**	284	Cold-acclimation-specific protein (CAS)
MSGI1_96533	11	5	**2580 (7)**	16	Cold acclimation-specific protein CAS)

^† ^Top 10 most abundant protein-coding transcripts selected from each library are highlighted in bold. Numbers enclosed in parenthesis represent rank based on transcript frequency for the top 10 most abundant protein-coding transcripts in each library.

We also identified putative housekeeping genes (HKGs) that showed little variation in expression but were expressed at relatively high levels. To identify HKGs, we first selected genes with an average RPKM-normalized transcript count greater than 10. Next, we selected the top 300 genes with the lowest coefficient of variation (CV = standard deviation/mean) (Additional file 14) [13]. These HKGs may be useful as reference genes in qRT-PCR or other experiments to normalize gene expression levels across different conditions [51].

### Identification of differentially expressed genes

We used a MA-plot-based method with a random sampling model in a DEGSeq program [52] to identify genes differentially expressed between stems of alfalfa genotypes 708 and 773. A total of 3,838 and 4,428 genes were differentially expressed between ES and PES tissues of genotypes 708 and 773, respectively (p < 0.001, FDR < 0.025, ≥ 2-fold difference) (Additional files 15 and 16). Among the genes that were differentially expressed between ES and PES internodes, 849 genes were detected in internodes of both genotypes. In addition, a total of 8,883 and 4,799 genes were differentially expressed between genotypes 708 and 773 within ES and PES internodes, respectively (p < 0.001, FDR < 0.025, ≥ 2-fold difference) (Additional files 17 and 18). Of the genes that were differentially expressed between the two genotypes, 2,422 were detected in both ES and PES internodes. Among the 13,797 differentially expressed genes identified in four pair-wise comparisons of ES and PES internodes of the two genotypes, about 85% were ubiquitously expressed in all four libraries (RPKM-normalized transcript count > 0 in all 4 libraries), about 5.5% were expressed in three libraries, about 9.6% were expressed in two libraries, and 16 genes were expressed in only one library (Additional file 19). These results suggest that stem tissue internodes in alfalfa may be characterized on the basis of differential expression of ubiquitous genes or tissue/genotype-specific expression of selected genes as shown in previous studies with other species [12,13,40]. SNPs were detected in 700 differentially expressed genes. Interestingly, about 14% of these SNP-harboring differentially expressed genes were cell wall-related genes.

**Figure 5:**
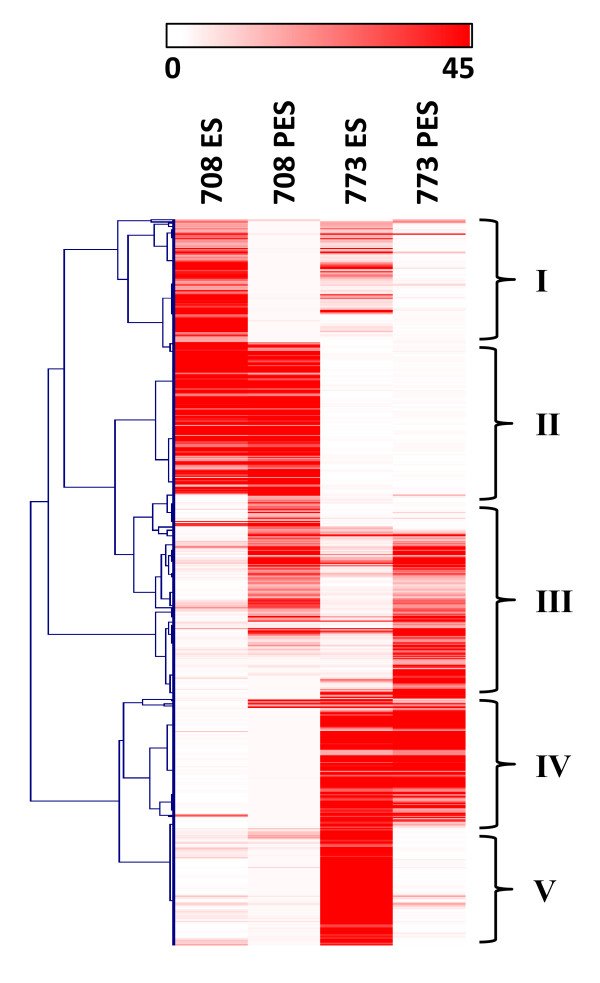
**Hierarchical clustering analysis of the top 200 most differentially expressed genes selected from pair-wise comparisons**. Pair-wise comparisons of gene expression were made between stem tissues (ES, PES) in alfalfa genotypes 708 and 773. The RPKM-normalized expression counts for each gene in each library are represented by intensity of the red color on a 0 to 45 scale. Dark red (scale intensity 45) indicates genes with RPKM-normalized expression counts ≥ 45. See Methods for details. Groups I and III, genes differentially expressed in a tissue-specific manner; Groups II and IV, genes differentially expressed in a genotype-specific manner; and Group V, genes differentially expressed in both a genotype- and tissue-specific manner. A complete list of the genes, RPKM-normalized expression counts, and corresponding MapMan functional categories are provided in Additional file 20.

To illustrate the differential expression of genes detected in the stem internodes of 708 and 773, we generated a heatmap of RPKM-normalized transcript counts for the top 200 most differentially expressed genes in each pair-wise comparison (Figure 5, Additional file 20). Groups I and III in Figure 5 contain genes that were differentially expressed in a tissue-specific manner which suggests their role in alfalfa stem development. For example, one expansin and four pectin esterase genes included in group I were up-regulated in ES compared to PES internodes in both genotypes. These genes are involved in cell wall loosening and cell elongation [53,54]. On the other hand, a putative alfalfa cellulose synthase gene, *IRREGULAR XYLEM 3 *(*IRX3*), included in group III (Figure 5) was up-regulated in PES internodes compared to ES in both genotypes. Several previous studies demonstrated xylem specific expression of *IRX3 *and its role in secondary cell wall development in *Arabidopsis *[55-57]. Groups II and IV in Figure 5 contain genes differentially expressed in a genotype-specific manner suggesting possible roles in the genotypic variation between stems of 708 and 773. For example, two extensin genes and a cellulose synthase gene (*CESA4*) included in group II were up-regulated in genotype 708 compared to 773 in both ES and PES internodes. These genes may be responsible for the higher cellulose content in stem internodes of genotype 708 compared to 773. Group V in Figure 5 contains genes differentially expressed in both a genotype- and tissue-specific manner.

**Figure 6:**
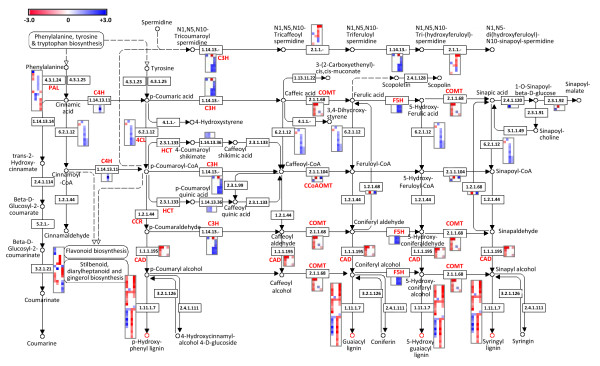
**Lignin pathway genes differentially expressed in stem tissues of two alfalfa genotypes**. Pair-wise comparisons were made between stem tissues (ES, PES) of genotypes 708 and 773. Columns in each heatmap from left to right: Log_2_(708ES/773ES), Log_2_(708PES/773PES), Log_2_(708PES/708ES), and Log_2_(773PES/773ES). The rows in each heatmap represent lignin gene sequences identified in MSGI 1.0. The Log_2 _expression ratio values were false color-coded using a scale of -3 to 3. The intensity of blue and red indicates the degree of up- and down-regulation of the corresponding lignin gene in the denominator in each column mentioned above. The red and blue color saturates at -3 and 3, respectively. See Methods for details. The heatmaps generated were inserted next to the corresponding lignin gene in the lignin biosynthetic pathway diagram downloaded from the KEGG pathway database http://www.genome.jp/kegg/pathway/map/map00940.html. PAL, phenylalanine ammonia-lyase; C4H, cinnamate-4-hydroxylase; 4CL, 4-coumarate-CoA ligase; HCT, hydroxycinnamoyltransferase; C3H, p-coumarate 3-hydroxylase; CCoAOMT, caffeoyl-CoA 3-O-methyltransferase; CCR1, cinnamoyl-CoA reductase 1; F5H, ferulate 5-hydroxylase; COMT, caffeic acid O-methyltransferase; CAD, cinnamyl-alcohol dehydrogenase.

Lignin content in alfalfa stems affects the quality of alfalfa as a forage crop and biomass feedstock. Lignin is indigestible and reduces cell wall digestibility in ruminants [58-60]. In addition, the pre-treatment process to remove lignin is one the most costly steps of cellulosic ethanol production [61-64]. Over multiple environments, alfalfa genotype 773 consistently showed higher cell wall lignin content in stems compared to genotype 708 (Figure 1) suggesting differences in the genetics of lignin biosynthesis. In an effort to identify key genes responsible for differences in cell wall properties in stems of genotypes 708 and 773, we identified lignin (phenylpropanoid) pathway genes among the 13,797 genes detected (Additional file 21). Next, we generated a heatmap of gene expression ratios for each selected lignin pathway gene for each pair-wise comparison (see Methods for details). The heatmaps generated were inserted into the lignin biosynthetic pathway (Figure 6). As expected, numerous lignin pathway genes were up-regulated in PES compared to ES internodes (Figure 6, Additional file 21). We also identified lignin genes differentially expressed between the two alfalfa genotypes. For example, several *CAD *and *COMT *genes were up-regulated in 773 compared to 708 especially in ES internodes (Figure 6, Additional file 21). These genes may contribute to difference in lignin content in cell walls of stems of genotypes 708 and 773.

A previous study [25] and the current study both suggest significant genotypic variation for gene expression in alfalfa stem internodes. To identify genes involved in general stem development (ES vs. PES internodes) independent of genotypic variation in gene expression, we selected a subset of alfalfa genes differentially expressed between ES and PES internodes in both genotype 708 and genotype 773 (p < 0.001, FDR < 0.025, ≥ 2-fold difference). A total of 594 genes were identified by further selecting genes with similar differential expression patterns in both genotypes [Log_2_(PES/ES) ≥ 1 or ≤ -1 in both genotypes] (Additional file 22). Among these genes, about 19% were cell wall-related genes. These genes included 5 cellulose synthase genes (a putative *IRX3*, two *CesA8s*, and two *COBRAs*) and six lignin pathway genes (three 4CLs and three F5Hs) that were up-regulated in PES compared to ES internodes in both genotypes (Additional file 22). In *Arabidopsis*, *IRX3*, *CesA*8 (*IRX1*) and *COBRA *genes are involved in cellulose biosynthesis during secondary cell wall development [46,55-57,65,66]. The gene families that were significantly over-represented among genes up-regulated in PES compared to ES internodes in both genotypes (Fisher's exact test with Bonfferoni correction with z-value cutoff of 1) included arabinogalactan protein (AGP), arginosuccinate synthase, metal handling, and transporter (sucrose, amino acids, and phosphate) families (Additional file 23). The gene families significantly over-represented among genes up-regulated in ES compared to PES internodes in both genotypes included invertase, pectin esterase, simple phenol, gibberellin-responsive, cold-responsive, lipid transfer protein (LTP), and GDSL-motif lipase families (Additional file 23). Cell wall family genes were over-represented among genes up-regulated in both ES and PES Internodes.

Assimilated photosynthetic carbon is translocated primarily as sucrose in higher plants [67]. Membrane-bound, energy dependent, H^+^-symporting sucrose transporters (*SUC *or *SUT *proteins) play an essential role in sucrose uptake in sink tissues and sucrose release in source tissues [67]. In this study, members of the sucrose transporter gene family were over-represented among genes up-regulated in PES compared to ES internodes in both genotypes (Additional file 23). Previous studies showed that the expression of sucrose transporter genes was developmentally regulated in plants [68-73]. For example, sucrose transporter genes were up-regulated during secondary cell wall synthesis in developing cotton fibers [73]. In this study, we identified five putative sucrose transporters (*MsSUCs*) that were up-regulated in PES compared to ES internodes in both genotypes (Additional file 22, Additional file 24). As stem development progresses from ES to PES, sink strength may also increase due to secondary cell wall formation in secondary xylem. The up-regulation of *MsSUCs *in PES internodes may be in response to increased demand for sucrose and UDP-glucose to support cellulose synthesis during secondary cell wall formation. Consistent with this explanation is our finding that three sucrose synthase (*MsSuSy*) genes were up-regulated in PES compared to ES internodes in both genotypes. Sucrose synthase provides the UDP-glucose needed for cellulose synthesis [74,75]. In addition to their roles in providing sucrose and UDP-glucose for cellulose synthesis in secondary cell walls, *MsSUCs *and *MsSuSy *genes, respectively, may play important roles in modulating sugar sensing and signal transduction pathways during stem development in alfalfa [76].

In addition to the *SUC *transporter gene family, we also found that the phosphate (Pi) transporter gene family was over-represented among genes up-regulated in PES compared to ES internodes in both genotypes (Additional file 23). We identified six putative *PHOSPHATE1 *(*PHO1*) genes up-regulated in PES compared to ES internodes in both genotypes (Additional file 22, Additional file 24). In *Arabidopsis *root epidermal and cortical cells, PHO1 is involved in Pi loading into the xylem [77,78]. A recessive mutation in *PHO1 *in *Arabidopsis *resulted in reduced Pi loading into xylem [77,78]. *PHO1 *is expressed predominantly in roots and up-regulated under conditions of Pi starvation [78-80]. A recent study in *Arabidopsis *showed that the expression of *PHO1 *was modulated by *WRKY6 *and *WRKY42 *transcription factors in response to low Pi [81]. Up-regulation of *PHO1 *genes in PES may be needed to meet the requirements of Pi uptake and redistribution during cellulose synthesis in secondary cell walls. For example, the fructose released by *SuSy *(sucrose -> UDP-glucose + fructose) needs to be phosphorylated to be recycled by sucrose phosphate synthase (SPS).

The plant hormone auxin is a key regulator of plant growth and development [82]. In addition to its role in cell wall loosening and cell elongation [82], auxin also regulates vascular tissue differentiation and patterning in plants [82-85], secondary xylem development in trees [86,87], and fiber development in cotton [88]. Indole-3-acetic acid (IAA), the major auxin species, is made in the shoot apex and transported to the root apex [82]. Directional auxin transport is mainly controlled by the coordinated action of auxin influx (*AUX1*) and efflux (*PIN*) carrier complexes [82]. *AUX1*, an amino acid permease-like membrane protein, was originally identified after screening for auxin resistant mutants [89]. In *Arabidopsis*, *AUX1 *was preferentially expressed in xylem compared to phloem and nonvascular tissues of the root-hypocotyl [90]. *Arabidopsis AUX1 *mutants showed a reduction in lateral root formation [91] but enhanced root generation in shoot regeneration media [92]. In addition, disruption of polar auxin transport in *Arabidopsis *resulted in ectopic vascular differentiation in leaves [93]. Polarized auxin transport is essential for providing directional and positional signals for various developmental processes such as apical dominance, organ development, tropic growth, embryogenesis and vascular development [82-85,94-98]. In this study, the amino acid transporter gene families, which include *AUX1 *genes, were over-represented among genes up-regulated in PES compared to ES internodes in both genotypes (Additional file 23). A total of 5 putative *AUX1 *genes were up-regulated in PES (Additional file 22, Additional file 24).The up-regulation of *AUX1 *in PES internodes of alfalfa and the resultant increase in auxin uptake may play an important role in the formation of secondary xylem. A recent study in trees suggested that the radial auxin concentration gradient in cell types of secondary xylem modulates the expression of a small number of key genes that regulate secondary xylem development [87].

In addition to transporter family genes that were differentially expressed between ES and PES internodes of both genotypes, we also identified transporter family genes that were differentially expressed between genotypes. For example, several sugar (glucose, hexose, and sucrose) transporters and *AUX1 *genes were up-regulated in 708 compared to 773 in both ES and PES internodes (Additional file 24, Additional file 25). These transporters may play a role in the higher cellulose and sugar (galactose and mannose) content in stem internodes of genotype 708 compared to 773 (Table 1). We also identified numerous transporter families that were up-regulated in both ES and PES internodes of 773 compared to 708. Among these up-regulated transporter families were the multi-drug toxic efflux carrier (MATE) and ATP-binding cassette (ABC) transporter families (Additional file 24, Additional file 25). Recent studies suggest that monolignols synthesized in the cytoplasm are transported across the plasma membrane into the cell wall matrix where they are polymerized into lignin [99,100]. However, little is known about the transport mechanism. Previous studies have suggested that monolignol transport across the plasma membrane may involve passive diffusion [101] or may be mediated by membrane-bound transporters [102]. Genes in the MATE transporter family may be good candidates for monolignol transporters because they are involved in transport of proanthocyanidin precursors across the tonoplast in *Arabidopsis *and *M. truncatula *[103,104]. A role for ABC transporters in monolignol transport across the plasma membrane has been postulated because of their known role in transporting various secondary metabolites in plants [99,100,105,106]. Additional research will be required to determine whether the up-regulation of the MATE efflux carrier and ABC transporter families in stems of 773 (high-lignin) compared to 708 (low lignin) (Additional file 24, Additional file 25) contributes to the higher lignin content in cell walls of 773 (Table 1). The up-regulated MATE efflux carrier and ABC transport genes that we identified provide a list of candidate genes that will be useful in future research to evaluate the involvement of these gene families in monolignol transport.

## Conclusion

This study represents the first application of RNA-Seq technology for genomic studies in alfalfa. Our results demonstrate that RNA-Seq can be successfully used for gene identification, polymorphism detection and transcript profiling in alfalfa. Using RNA-Seq has several advantages over other technologies, especially for non-model species with few genomic resources such as alfalfa. Unlike hybridization-based technologies such as microarrays, RNA-Seq does not require pre-existing sequence information and, as shown in this study, RNA-Seq can integrate multiple tasks in a single pipeline saving time and money. The integrated approach used in this study can be applied to other non-model species. The newly built alfalfa gene index (MSGI 1.0), and the SNPs, SSRs and candidate genes identified in this study will be a valuable resource for advancing genetic/genomic research in alfalfa and eventually for improving alfalfa as a forage crop and cellulosic ethanol feedstock.

## Methods

### Plant materials and cell wall analysis

Alfalfa [*Medicago sativa *(L) subsp. *sativa*] genotypes 708 and 773 were selected from a population (UMN 3097) created by mixing seeds from six commercial alfalfa cultivars (5312, Rushmore, Magnagraze, Wintergreen, Windstar and WL 325HQ) as previously described [25]. The alfalfa clonal lines 708 and 773 were propagated from cuttings and grown in the greenhouse. The greenhouse experiments consisted of three replicates arranged in a randomized complete block design. For each replicate, there were eight plants of each clone in individual pots. For cell wall analysis, stem internodes tissues were harvested at full bloom and plant material for analysis was composited within each replicate (2 blocks × 3 reps = 6 data points per genotype). Cell wall analysis was performed in duplicate as previously described [25]. An analysis of variance was done to test if the means (g kg^-1 ^cell wall) for cell wall components of the two genotypes were equal (Table 1). For RNA-Seq, ES and PES internodes were harvested as previously described [25].

### RNA extraction, cDNA library preparation and sequencing

Total RNA was purified from three replicates of elongating and post-elongation stem internodes of genotypes 708 and 773 using the CTAB based protocol previously described [40]. Contaminating genomic DNA was removed from each RNA sample using the DNA-*free*™ kit following the manufacturer's recommendations http://www.ambion.com. An equal amount of total RNA was pooled from each replicate for each stem tissue sample. RNA samples were quantified using Quant-iT™ RiboGreen^® ^RNA Reagent http://www.invitrogen.com and the RNA integrity was checked with RNA6000 Nano Assay using the Agilent 2100 Bioanalyzer™ (Agilent Technologies, Palo Alto, CA). cDNA library preparation and sequencing reactions were conducted in the Biomedical Genomics Center, University of Minnesota. Illumina library prep, clustering and sequencing reagents were used throughout the process following the manufacturer's recommendations http://www.illumina.com. Briefly, mRNAs were purified using poly-T oligo-attached magnetic beads and then fragmented. The first and the second strand cDNAs were synthesized and end repaired. Adaptors were ligated after adenylation at the 3'-ends. After gel purification, cDNA templates were enriched by PCR. cDNA libraries were validated using a High Sensitivity Chip on the Agilent2100 Bioanalyzer™ (Agilent Technologies, Palo Alto, CA). The cDNA library was quantified using PicoGreen Assay and by qPCR. The samples were clustered on a flow cell using the cBOT. After clustering, the samples were loaded on the Illumina GA-II machine. The samples were sequenced using a single read with 76 cycles. Initial base calling and quality filtering of the Illumina GA-II image data were performed using the default parameters of the Illumina GA Pipeline GERALD stage http://www.illumina.com. Additional filtering for homopolymers and read size (< 75 bp) was performed using custom written code.

For RNA-Seq using the GS FLX Titanium platform http://www.454.com, mRNA was reverse transcribed with SuperScript III reverse transcriptase http://www.invitrogen.com using dT15VN2 primer. cDNA was synthesized using *E. coli *DNA Ligase, *E. coli *DNA polymerase I and *E coli *RNaseH. cDNA was then fragmented by sonication. The cDNA was then used for 454 sstDNA preparation in the "GS20 DNA Library Preparation" step2 http://www.454.com. The rest of the library preparation and the 454 sequencing procedures were performed following the manufacturer's recommendations http://www.454.com. Standard post-run and bioinformatics processing on the 454 platform to determine reads that passed various quality filters were also performed following the manufacturer's recommendations http://www.454.com.

### *de novo *transcriptome assembly

The Velvet algorithm [32] was used for *de novo *assembly of the 198,861,304 Illumina reads (76 bp). During the *de novo *assembly using the Velvet program, short EST reads were first hashed based on a predefined hash length in base pairs (*k*-mer length). Next, the contigs were built based on a series of overlapping *k*-mers using de Brujin graphs [32]. In general, longer *k*-mers increase transcript contiguity (longer transcript length) and specificity (less spurious overlaps) but decrease diversity (smaller number of contigs) compared to shorter *k*-mers [32]. To optimize our Velvet assembly toward higher transcript contiguity and specificity, we tested a series of *k*-mers (31, 37, 41, 47, 51, 57, 61, 63, 65) for *de novo *assembly of short EST reads (Additional file 26). We used the median contig length (N50) generated for each *k*-mer as an indicator of the transcript contiguity of *de novo *assembly. As *k*-mer values increased from 31 to 61, N50 values increased to a value of 289 reflecting increased efficiency of *de novo *assembly. The N50 values declined significantly at *k*-mer values above 61 (Additional file 26). On the basis of these results, we used a *k*-mer value of 61 for *de novo *assembly of alfalfa EST reads.

### Alfalfa Gene Index assembly

The alfalfa gene index (MSGI 1.0) was built following the Gene Index Assembly protocol previously described [39,40]. The gene ontology (GO) functional classes and pathways for each sequence in MSGI 1.0 were assigned based on *Arabidopsis *GO SLIM and pathway annotation ftp://ftp.arabidopsis.org/home/tair/Ontologies/. For GO characterization, the unique sequences in MSGI 1.0 were compared with the *Arabidopsis *proteome using the BlastX program with e-value cutoff of 1e-10. Top protein matches from *Arabidopsis *sequences were assigned to each of the MSGI 1.0 sequences. The MapMan gene functional classification system [41] was assigned to each sequence in MSGI 1.0 following the method previously described [31]. The functional class over-representation analysis was performed using PageMan [107] as previously described [25,31].

### Polymorphism detection

The MISA program [42] was used to detect simple sequence repeats (SSRs) among sequences in MSGI 1.0. The minimum number of nucleotide repeats specified during SSR analysis was 20, 10, 7, 5, 5, and 5 for mono-, di-, tri-, tetra-, penta-, and hexanucleotide repeats, respectively. The maximum number of bases interrupting 2 SSRs in a compound microsatellite was set at 100 bp. The primers spanning each SSR were designed using the default parameter of the Primer3 program [44].

For SNP detection, the Illumina GA-II reads were mapped to the sequences in MSGI 1.0 using the Maq program [45]. Next, the coverage and nucleotide differences were extracted using the pileup command of the Maq program. The pileup output was further compiled for genotypes 708 and 773 with custom written script using filtering based on coverage and quality scores. Custom written script was used for additional sorting and filtering of the pileup output based on a nucleotide depth cutoff of 10 for each SNP.

### Digital gene expression analysis

For digital gene expression analysis, the raw digital gene expression counts were measured by quantifying the number of Illumina GA-II reads that were mapped to the reference sequences (MSGI 1.0 or Mt3.0 cds) using the bowtie program [47]. The best-match option with a maximum of 3 nucleotide mismatches was used (-v 3 --best). The raw digital gene expression counts were normalized using the RPKM (reads/Kb/Million) method [1,48]. Custom written scripts were used to summarize the bowtie output from the raw digital expression counts and the RPKM-normalized expression counts. To identify differentially expressed genes, an expression profile matrix was built representing the digital gene expression count for each gene in each library, then imported into the DEGSeq program [52]. A DEGSeq program that utilized a MA-plot-based method with random sampling model was used to identify differentially expressed genes in each pair-wise comparison (p < 0.001, FDR < 0.025, ≥ 2-fold difference). Heatmaps based on hierarchical cluster analysis [108] of RPKM-normalized expression counts (Figure 5, Additional file 25) and expression ratios (Figure 6) were generated using MultiExperiment Viewer http://www.tm4.org/mev/.

In a previous study, we generated GeneChip data for ES and PES internodes of alfalfa genotypes 252 and 1283 [25]http://www.ncbi.nlm.nih.gov/geo/query/acc.cgi?acc=GSE13602. To compare the digital gene expression data generated using MSGI 1.0 and Mt3.0 cds sequences with the previously generated GeneChip data [25], we first compared two *Medicago *reference sequences (MSGI 1.0 and Mt3.0 cds) with *Medicago *GeneChip probe set consensus sequences using the Blastn program (e-value cutoff of 1e-10). Top sequence matches from the *Medicago *GeneChip probe sets were assigned to each RNA-Seq reference sequence. Next, we selected from GeneChip data and RNA-Seq data a subset of genes involved in general stem development independent of genotypic variation in gene expression (Log_2_(PES/ES) ≥ 1 or ≤ -1 in both genotypes). Genes that were commonly selected between RNA-Seq and GeneChip data were identified based on sequence homology. Log_2_(PES/ES) values from the RNA-Seq data generated using MSGI 1.0 and Mt3.0 cds as reference sequences were compared with Log_2_(PES/ES) values from the GeneChip data (Figure 4A, 4B).

To compare the digital gene expression data generated using MSGI 1.0 and Mt3.0 cds sequences with the qRT-PCR data, we first compared two *Medicago *reference sequences (MSGI 1.0 and Mt3.0 cds) using the the Blastn program (e-value cutoff of 1e-10). Top sequence matches from the Mt3.0 cds were assigned to each MSGI 1.0 sequence. Primers for qRT-PCR were designed based on the MSGI 1.0 sequences (Additional file 10). Log ratio values from the RNA-Seq data generated using MSGI and Mt3.0 cds as reference sequences were compared with ΔΔC_T _values obtained from the qRT-PCR data (Figure 4C, 4D).

### SNP genotyping

The SNP genotyping was conducted in the Biomedical Genomics Center, University of Minnesota. Briefly, a total of 55 SNPs predicted between genotypes 708 and 773 were randomly selected for validation by MALDI-TOF mass spectrometry using the iPLEX Gold spectrometry system http://www.sequenom.com. Genomic DNAs were purified from young leaves of genotypes 708 and 773 using DNeasy Plant Mini Kit http://www.qiagen.com. The multiplex assays were designed using Mass-ARRAY Assay Design 3.0 software and primers were obtained from IDT (Coralville, Iowa). Reactions (PCR, shrimp alkaline phosphatase treatment followed by extension) were performed according to iPLEX Gold method http://www.sequenom.com. Mass ARRAY workstation software (v. 3.3) was used to analyze the SNP genotyping results.

### Real-time quantitative RT-PCR (qRT-PCR)

A portion of the pooled total RNA used for the RNA-Seq analysis was used to make cDNAs for qRT-PCR. The first strand cDNA for each sample was made using random hexamers and Taqman Reverse Transcription Reagents (Applied Biosystems, CA) following the manufacturer's recommendations. Gene specific primers based on MSGI 1.0 sequences were subsequently designed using Primer Express (Applied Biosystems, CA) (Additional file 10). Samples and standards were run in triplicate on each plate and repeated on two plates using SYBR-Green PCR Master Mix (Applied Biosystems, CA) on a StepOnePlus™ Real-Time PCR System (Applied Biosystems, CA) following the manufacturer's recommendations. qRT-PCR was performed in a 20 μl reaction containing 4 μl ddH_2_O, 10 μl 2× PCR mix, 1 μl forward primer (1 μM), 1 μl reverse primer (1 μM), and 4 μl of template cDNA (5 ng/μl). The PCR conditions were as follows: two minutes of pre-incubation at 50°C, 10 minutes of pre-denaturation at 94 °C, 40 cycles of 15 seconds at 95 °C and one min at 60 °C, followed by steps for dissociation curve generation (30 seconds at 95 °C, 60 seconds at 60 °C and 30 seconds at 95 °C). The StepOnePlus software (Applied Biosystems, CA) was used for data collection and analysis. Dissociation curves for each amplicon were carefully examined to confirm lack of multiple amplicons at different melting temperatures (Tms). Relative transcript levels for each sample were obtained using the "comparative C_T _method" [109] using the C_T _value of the 18S rRNA for each sample as a normaliser.

## Abbreviations

ES: elongating stem; PES: post-elongation stem; SNP: single nucleotide polymorphism; EST: expressed sequence tag; GO: gene ontology; cds: coding sequence; SSR: simple sequence repeat; RPKM: reads/Kb/Million; q-RT PCR: real-time quantitative RT-PCR; HKG: housekeeping gene; CV: coefficient of variation; CesA: cellulose synthase; PAL: phenylalanine ammonia-lyase; C4H: cinnamate-4-hydroxylase; 4CL: 4-coumarate-CoA ligase; HCT: hydroxycinnamoyl transferase; C3H: p-coumarate 3-hydroxylase; CCoAOMT: caffeoyl-CoA 3-O-methyltransferase; CCR1: cinnamoyl-CoA reductase 1; F5H: ferulate 5-hydroxylase; COMT: caffeic acid O-methyltransferase; CAD: cinnamyl-alcohol dehydrogenase; AGP: arabinogalactan protein; LTP: lipid transfer protein; LHB1B1: Photosystem II light harvesting complex gene; RBCS-1A: rubisco small subunit 1; SUC: sucrose transporter; SuSy: sucrose synthase; PHO1: PHOSPHATE 1; IAA: Indole-3-acetic acid; AUX1: auxin influx carrier; MATE: multi-drug toxic efflux carrier; ABC: ATP-binding cassette.

## Authors' contributions

SY and ZT performed the computational analysis involved in the *de novo *assembly, digital gene expression and SNP detection. CF performed the computational analysis involved in MSGI 1.0 assembly and SSR detection. SY and WX performed the computational analysis involved in the identification of differentially expressed genes. SY performed the computational analysis involved in the functional classification and over-representation analysis. SY conducted the qRT-PCR. JL identified the genotypes used in the study. HJ conducted the cell wall analysis of the alfalfa genotypes. All authors contributed to the analysis of results and writing of the manuscript. All authors read and approved the final manuscript.

## Supplementary Material

Additional file 1 ***de novo *assembly of alfalfa Illumina GA-II EST reads**. A fasta file containing a total of 132,153 unique sequences generated after *de novo *assembly of Illumina GA-II EST reads derived from 4 cDNA libraries developed in this study. The Velvet program [32] with *k*-mer 61 was used for *de novo *assembly. Click here for file

Additional file 2 **Alfalfa Gene Index 1.0 (MSGI 1.0)**. A fasta file containing Alfalfa Gene Index 1.0 (MSGI 1.0) sequences. MSGI 1.0 contains a total of 124,025 unique sequences including 22,729 tentative consensus sequences (TCs), 22,315 singletons and 78,981 pseudo-singletons. The average length of the unique sequences in MSGI 1.0 is 384 bp (100 bp minimum and 6,956 bp maximum) with more than 10,000 sequences larger than 800 bp. The total base count of the sequences in MSGI 1.0 is 47,628,953 bp. Unfortunately, the current pipe line of the DFCI gene index database http://compbio.dfci.harvard.edu/tgi/ is not suited for short reads (personal communication with a DFCI Gene Index staff). The Gene Index Project team has indicated that it plans to address this issue soon. When a gene index database is established for alfalfa, MSGI1.0 will be uploaded to the DFCI gene index database. Click here for file

Additional file 3 **Functional classification and annotation of sequences in the Alfalfa Gene Index 1.0 (MSGI 1.0)**. A table listing Gene ontology (GO), pathway, MapMan functional classes and gene annotation for sequences in the Alfalfa Gene Index 1.0 (MSGI 1.0). Click here for file

Additional file 4 **Simple sequence repeats (SSRs) detected in MSGI 1.0**. A table listing SSR-containing sequence IDs, SSR types and position, and primers spanning each SSR for the sequences in the Alfalfa Gene Index 1.0 (MSGI 1.0). Click here for file

Additional file 5 **Single nucleotide polymorphisms (SNPs) predicted between alfalfa genotypes 708 and 773**. A table listing SNPs predicted between alfalfa genotypes 708 and 773 including SNP-containing sequence ID, SNP type, SNP position and depth in each genotype. Click here for file

Additional file 6 **Validation of SNPs predicted between alfalfa genotypes 708 and 773 using RNA-Seq data**. A table showing SNP validation results. A total of 55 SNPs were randomly selected to genotype genomic DNAs purified from the genotypes 708 and 773 by MALDI-TOF mass spectrometry using the iPLEX Gold spectrometry system http://www.sequenom.com. In addition to genotypes 708 and 773, we also genotyped 51 additional alfalfa (*M. sativa*) genotypes selected from different populations of *M. sativa ssp. sativa *or *M. sativa ssp. falcata*. Click here for file

Additional file 7 **Functional classes over- or under-represented among SNP-harboring genes**. A figure showing the functional class over-representation analysis conducted for SNP-harboring genes. Functional classes that are over- or under-represented among SNP-harboring genes were identified using the PageMan over-representation analysis module. The z-vlaues for significant classes identified after Fisher's exact test with Bonferroni correction (z-value cutoff of 1) were false color coded using a scale of -5 to +5. The intensity of blue and red indicate the degree of over- and under-representation of the corresponding class, respectively. Click here for file

Additional file 8 **Allelic variations (SNPs) detected within genotype 708**. A table listing a total of 287,555 allelic variations (SNPs) detected within genotype 708 using minimum SNP depth cutoff of 10. Click here for file

Additional file 9 **Allelic variations (SNPs) detected within genotype 773**. A table listing a total of 168,966 allelic variations (SNPs) detected within genotype 773 using minimum SNP depth cutoff of 10. Click here for file

Additional file 10 **qRT-PCR validation of RNA-Seq data generated by two reference sequences (MSGI 1.0 and Mt3.0 cds)**. A table showing the source data used to generate Figure 4. The table contains MSGI 1.0 and Mt3.0 cds IDs of genes used for qRT-PCR, qRT-PCR and RNA-Seq data generated by two reference sequences (MSGI 1.0 and Mt3.0 cds), and primers used for qRT-PCR. Click here for file

Additional file 11 **An expression profile matrix for each library showing digital gene expression count of each gene in MSGI 1.0**. A table showing the digital gene expression counts of each gene in MSGI 1.0 for ES and PES internodes of alfalfa genotypes 708 and 773. The raw expression counts generated by bowtie program were normalized using the RPKM method [1,48]. Click here for file

Additional file 12 **Top 500 most abundant transcripts in each library**. A table showing the RPKM-normalized digital gene expression counts and MapMan functional classes for the top 500 most abundant transcripts selected in each library. Click here for file

Additional file 13 **Functional classes over- or under-represented among the top 500 most abundant transcripts in each library**. A figure showing the results from functional class over-representation analysis for the top 500 most abundant transcripts in ES and PES internodes of alfalfa genotypes 708 and 773. For details, see the description for additional file 7. Click here for file

Additional file 14 **300 housekeeping genes selected**. A table listing 300 housekeeping genes (HKGs) with relatively high levels of expression. To identify these HKGs, we first selected genes with an average RPKM-normalized transcript count greater than 10. Next, we selected the top 300 genes from the list with the lowest coefficient of variation (CV = standard deviation/mean). The RPKM-normalized expression counts, MapMan functional class and description for each HKG selected are also presented in the table. Click here for file

Additional file 15 **Genes differentially expressed between ES and PES internodes of alfalfa genotype 708**.A table listing 3,838 genes differentially expressed between ES and PES internodes of alfalfa genotype 708 in MSGI 1.0. We used a MA-plot-based method with random sampling model in a DEGSeq program to select these genes (p-value < 0.001, FDR < 0.025, ≥ 2-fold difference). RPKM-normalized expression counts, log ratios, z-scores, p-values, and q-values for each gene selected are also presented in the table. Click here for file

Additional file 16 **Genes differentially expressed between ES and PES internodes of alfalfa genotype 773**. A table listing 4,428 genes differentially expressed between ES and PES internodes of alfalfa genotype 708 in MSGI 1.0. For details, see the description for additional file 11. Click here for file

Additional file 17 **Genes differentially expressed between alfalfa genotypes 708 and 773 in ES internodes**.A table listing 8,883 genes differentially expressed between alfalfa genotypes 708 and 773 in ES internodes in MSGI 1.0. For details, see the description for additional file 11. Click here for file

Additional file 18 **Genes differentially expressed between alfalfa genotypes 708 and 773 in PES internodes**. A table listing 4,799 genes differentially expressed between alfalfa genotypes 708 and 773 in PES internodes in MSGI 1.0. For details, see the description for additional file 11. Click here for file

Additional file 19 **Genes differentially expressed in ES and PES internodes of alfalfa genotypes 708 and 773**. A table listing 13,797 genes differentially expressed in ES and PES internodes of alfalfa genotypes 708 and 773 in MSGI 1.0. Genes selected in additional files 15, 16, 17 and 18 were combined together to produce this table. The RPKM-normalized expression counts, MapMan functional class and description for each gene selected are also presented in the table. Click here for file

Additional file 20 **Top 200 most differentially expressed genes in each pair-wise comparison**. A table that lists 657 genes that were generated after combining the top 200 most differentially expressed genes selected in each pair-wise comparison of gene expression between ES and PES internodes of genotypes 708 and 773. This table is a data source for Figure 5. The RPKM-normalized expression counts, MapMan functional class and description for each gene selected are also presented in the table. Click here for file

Additional file 21 **Phenylpropanoid (lignin) pathway genes differentially expressed in ES and PES internodes of alfalfa genotypes 708 and 773**. A table listing phenylpropanoid (lignin) pathway genes differentially expressed in ES and PES internodes of alfalfa genotypes 708 and 773 (p-value < 0.001, FDR,0.025, ≥ 2-fold difference). This table is a data source for Figure 6. The log ratios from each pair-wise comparison, EC number, and enzyme ID for each gene selected are also presented in the table. Click here for file

Additional file 22 **Candidate genes identified in 708 and 773 that may be involved in general stem development independent of genotypic variation in gene expression**. A table listing 594 genes potentially involved in general stem development independent of genotypic variation in gene expression in alfalfa (Log2(PES/ES)≥1 or ≤-1 in both genotypes 708 and 773). The RPKM-normalized expression counts, log ratios, MapMan functional class and description for each gene selected are also presented in the table. Click here for file

Additional file 23 **Functional classes over- or under-represented among genes involved in general stem development independent of genotypic variation in alfalfa**. A figure showing the functional class over-representation analysis for genes involved in general stem development independent of genotypic variation in alfalfa (Log2(PES/ES)≥1 or ≤-1 in both genotypes 708 and 773). "Up in PES" and "Up in ES" indicate genes up-regulated in PES and ES internodes in both genotypes, respectively. For details, see the description for additional file 7. Click here for file

Additional file 24 **Putative transporter genes differentially expressed in ES and PES internodes of alfalfa genotypes 708 and 773**. A table listing 478 transporter genes in ES and PES internodes of alfalfa genotypes 708 and 773 in MSGI 1.0. The RPKM-normalized expression counts, log ratios from each pair-wise comparison, MapMan functional class and description for each transporter gene selected are also presented in the table. Click here for file

Additional file 25 **Hierarchical clustering analysis of selected transporter genes differentially expressed between 708 and 773 in both ES and PES internodes**. A figure showing a heatmap for 42 transporter genes differentially expressed between 708 and 773 in both ES and PES internodes (p < 0.001, FDR < 0.025, ≥ 2-fold difference). The RPKM-normalized expression counts for each gene in each library are represented by the intensity of the red color on a 0 to 22 scale. Dark red (scale intensity 22) indicates genes with RPKM-normalized expression counts ≥ 22. See Methods for details. A complete list of the transporter genes selected, RPKM-normalized expression counts, and corresponding MapMan functional categories are provided in Additional file 24. Click here for file

Additional file 26 **Optimization of *de novo *assembly of Illumina GA-II EST reads with a series of *k*-mers using the Velvet program **[32]. A figure showing the median sequence length of the contigs (y-axis) for a series of *k*-mers (31, 37, 41, 47, 51, 57, 61, 63, 65) tested using the Velvet program. *k*-mer 61 produced the longest median sequence length. Click here for file

## References

[B1] Mortazavi A Williams BA McCue K Schaeffer L Wold B Mapping and quantifying mammalian transcriptomes by RNA-Seq Nat Methods 2008 5 7 621 628 10.1038/nmeth.1226 18516045 PMC13303166

[B2] Lister R Gregory BD Ecker JR Next is now:new technologies for sequencing of genomes, trancriptomes, and beyond Curr Opin Plant Biol 2009 12 107 118 10.1016/j.pbi.2008.11.004 19157957 PMC2723731

[B3] Marguerat S Bähler J RNA-seq: from technology to biology Cell Mol Life Sci 2010 67 569 579 10.1007/s00018-009-0180-6 19859660 PMC2809939

[B4] Wilhelm BT Landry J-R RNA-Seq-quantitative measurement of expression through massively parallel RNA-Sequencing Methods 2009 48 249 257 10.1016/j.ymeth.2009.03.016 19336255

[B5] Wang Z Gerstein M Snyder M RNA-Seq: a revolutionary tool for transcriptomics Nat Rev Genet 2009 10 57 63 10.1038/nrg2484 19015660 PMC2949280

[B6] Bruno VM Wang Z Marjani SL Euskirchen GM Martin J Sherlock G Snyder M Comprehensive annotation of the trancriptome of the human fungal pathogen *Candida albicans *using RNA-seq Genome Res 2010 20 1451 1458 10.1101/gr.109553.110 20810668 PMC2945194

[B7] Rounsley SD Last RL Shotguns and SNPS: how fast and cheap sequencing is revolutionizing plant biology Plant J 2010 61 922 927 10.1111/j.1365-313X.2009.04030.x 20409267

[B8] Howard BE Heber S Towards reliable isoform quantification using RNA-SEQ data BMC Bioinformatics 2010 11 Suppl 3 56 20438653 10.1186/1471-2105-11-S3-S6 PMC2863065

[B9] Marioni JC Mason CE Mane SM Stephens M Gilad Y RNA-seq: An assessment of technical reproducibility and comparison with gene expression arrays Genome Res 2008 18 1509 1517 10.1101/gr.079558.108 18550803 PMC2527709

[B10] Weber APM Weber KL Carr K Wilkerson C Ohlrogge JB Sampling the Arabidopsis transcriptome with massively parallel pryrosequencing Plant Physiol 2007 144 1 32 42 10.1104/pp.107.096677 17351049 PMC1913805

[B11] Wall PK Leebens-Mack J Chanderbali AS Barakat A Wolcott E Liang H Landherr L Tomsho LP Hu Y Carlson JE Ma H Schuster SC Soltis DE Soltis PS Altman N dePamphilis CW Comparison of next generation sequencing technologies for transcriptome characterization BMC Genomics 2009 10 347 10.1186/1471-2164-10-347 19646272 PMC2907694

[B12] Libault M Farmer A Joshi T Takahashi K Langley RJ Franklin LD He J Xu D May G Stacey G An integrated transcriptome atlas of the crop model *Glycine max*, and its use in comparative analyses in plants Plant J 2010 10 1111 10.1111/j.1365-313X.2010.04222.x 20408999

[B13] Severin AJ Woody JL Bolon YT Joseph B Diers BW Farmer AD Muehlbauer GJ Nelson RT Grant D Specht JE Graham MA Cannon SB May GD Vance CP Shoemaker RC RNA-Seq atlas of *Glycine max*: A guide to the soybean transcriptome BMC Plant Biology 2010 10 160 10.1186/1471-2229-10-160 20687943 PMC3017786

[B14] Lu T Lu G Fan D Zhu C Li W Zhao Q Feng Q Zhao Y Guo Y Li W Huang X Han B Functional annotation of the rice transcriptome at single-nucleotide resolution by RNA-seq Genome Res 2010 20 1238 1249 10.1101/gr.106120.110 20627892 PMC2928502

[B15] Barbazuk WB Emrich SJ Chen HD Li L Schnable PS SNP discovery via 454 transcriptome sequencing Plant J 2007 51 5 910 918 10.1111/j.1365-313X.2007.03193.x 17662031 PMC2169515

[B16] Cheung F Haas BJ Goldberg SMD May GD Xiao Y Town CD Sequencing *Medicago truncatula *expressed sequenced tags using 454 Life Sciences technology BMC Genomics 2006 7 272 10.1186/1471-2164-7-272 17062153 PMC1635983

[B17] Novaes E Drost DR Farmerie WG Pappas GJ Jr Grattapaglia D Sederoff RR Kirst M High-throughput gene and SNP discovery in *Eucalyptus grandis*, an uncharacterized genome BMC Genomics 2008 9 312 10.1186/1471-2164-9-312 18590545 PMC2483731

[B18] Bellin D Ferrarini A Chimento A Kaiser O Levenkova N Bouffard P Delledonne M Combining next-generation pyrosequencing with microarray for large scale expression analysis in non-model species BMC Genomics 2009 10 555 10.1186/1471-2164-10-555 19930683 PMC2790472

[B19] Collins LJ Biggs PJ Voelckel C Joly S An approach to transcriptome analysis of non-model organisms using short-read sequences Genome Informatics 2008 21 3 14 19425143

[B20] Wang W Wang Y Zhang Q Qi Y Guo D Global characterization of *Artemisia annua *glandular trichome transcriptome using 454 pyrosequencing BMC Genomics 2009 10 465 10.1186/1471-2164-10-465 19818120 PMC2763888

[B21] Trick M Long Y Meng J Bancroft I Single nucleotide polymorphism (SNP) discovery in the polyploidy *Brassica napus *using Solexa transcriptome sequencing Plant Biotech J 2009 7 334 346 10.1111/j.1467-7652.2008.00396.x 19207216

[B22] Michaud R Lehman WF Rumbaugh MD Hanson AA, Barnes DK, Hill RR Jr World Distribution and Historical Development Alfalfa and alfalfa improvement - Agronomy Monograph no. 29 1988 Madison, WI: ASA-CSSA-SSSA 25 91

[B23] National Agricultural Statistics Service 2009 http://www.nass.usda.gov On-line resource

[B24] Samac DA Jung H-JG Lamb JFS Minteer S Development of alfalfa (*Medicago sativa *L.) as a feedstock for production of ethanol and other bioproducts Alcoholic Fuels 2006 Boca Raton, FL: CRC Press 79 98

[B25] Yang SS Xu WW Tesfaye M Lamb JFS Jung H-JG VandenBosch KA Vance CP Gronwald JW Transcript profiling of two alfalfa genotypes with contrasting cell wall composition in stems using a cross-species platform: optimizing analysis by masking biased probes BMC Genomics 2010 11 323 20497574 10.1186/1471-2164-11-323 PMC2893600

[B26] Rumbaugh MD Caddel JL Rowe E Breeding and Quantitative Genetics Alfalfa and Alfalfa Improvement. ASA Monograph 29 1988 Madison, WI: American Society of Agronomy 777 808

[B27] Brummer EC Sledge MK Bouton JH Kochert G Phillips RL, Vasil IK Molecular Marker Analyses in Alfalfa and Related Species DNA-based markers in plants 2001 The Netherlands: Kluwer Academic 169 180

[B28] Julier B Flajoulot S Barre P Cardinet G Santoni S Huguet T Huyghe C Construction of two genetic linkage maps in cultivated tetraploid alfalfa (*Medicago sativa*) using microsatellite and AFLP markers BMC Plant Biol 2003 3 9 10.1186/1471-2229-3-9 14683527 PMC324403

[B29] Diwan N Bhagwat AA Bauchan GB Cregan PB Simple sequence repeat DNA markers in alfalfa and perennial and annual *Medicago *species Genome 1997 40 887 895 10.1139/g97-115 18464874

[B30] Sledge MK Ray IM Jiang G An expressed sequence tag SSR map of tetrapolid alfalfa (*Medicago sativa *L.) Theor Appl Genet 2005 111 980 992 10.1007/s00122-005-0038-8 16075206

[B31] Yang SS Xu WW Tesfaye M Lamb JFS Jung H-JG Samac DA Vance CP Gronwald JW Single-feature polymorphism discovery in the transcriptome of tetraploid alfalfa Plant Genome 2009 2 224 232 10.3835/plantgenome2009.03.0014

[B32] Zerbino DR Birney E Velvet: algorithms for *de novo *short read assembly using de Bruijn graphs Genome Res 2008 18 821 829 10.1101/gr.074492.107 18349386 PMC2336801

[B33] Gibbons JG Janson EM Hittinger CT Johnston M Abbot P Rokas A Benchmarking next-generation transcriptome sequencing for functional and evolutionary genomics Mol Biol Evol 2009 26 12 2731 2744 10.1093/molbev/msp188 19706727

[B34] Mizrachi E Hefer CA Ranik M Joubert F Myburg AA *de novo *assembled expressed gene catalog of a fast-growing Eucalyptus tree produced by Illumina mRNA-Seq BMC Genomics 2010 11 681 10.1186/1471-2164-11-681 21122097 PMC3053591

[B35] Garg R Patel RK Tyagi AK Jain M *de novo *assembly of chickpea transcriptome using short reads for gene discovery and marker identification DNA Research 2011 10.1093/dnares/dsq028 PMC3041503 21217129

[B36] Birol I Jackman SD Nielsen CB Qian JQ Varhol R Stazyk G Morin RD Zhao Y Hirst M Schein JE *de novo *transcriptome assembly with ABySS Bioinformatics 2009 25 21 2872 2877 10.1093/bioinformatics/btp367 19528083

[B37] Surget-Groba Y Montoya-Burgos JI Optimization of *de novo *transcriptome assembly from next-generation sequencing data Genome Research 2010 20 1432 1440 10.1101/gr.103846.109 20693479 PMC2945192

[B38] Oases http://www.ebi.ac.uk/~zerbino/oases (Jan 31st, website last accessed)

[B39] Quackenbush J Computational analysis of microarray data Nat Rev Genet 2001 2 418 427 10.1038/35076576 11389458

[B40] Yang SS Cheung F Lee JJ Ha M Wei NE Sze SH Stelly DM Thaxton P Triplett B Town CD Chen ZJ Accumulation of genome-specific transcripts, transcription factors and phytohormonal regulators during early stages of fiber cell development in allotetraploid cotton Plant J 2006 47 761 775 10.1111/j.1365-313X.2006.02829.x 16889650 PMC4367961

[B41] Thimm O Bläsing O Gibon Y Nagel A Meyer S Krüger P Selbig J Müller LA Rhee SY Stitt M MAPMAN: a user driven tool to display genomics data sets onto diagrams of metabolic pathways and other biological processes Plant J 2004 37 914 939 10.1111/j.1365-313X.2004.02016.x 14996223

[B42] Thiel T Michalek W Varshney RK Graner A Exploiting EST databases for the development and characterization of gene-derived SSR-markers in barley (*Hordeum vulgare *L.) Theor Appl Genet 2003 106 411 422 12589540 10.1007/s00122-002-1031-0

[B43] Ellis JR Burke JM EST-SSRs as a resource for population genetic analyses Heredity 2007 99 125 132 10.1038/sj.hdy.6801001 17519965

[B44] Rozen S Skaletsky H Krawetz S, Misener S Primer3 on the www for general users and for biologist programmers Bioinformatics Methods and Protocols: Methods in Molecular Biology 2000 Totowa, NJ: Humana Press 365 386 10.1385/1-59259-192-2:365 10547847

[B45] Li H Ruan J Durbin R Mapping short DNA sequencing reads and calling variants using mapping quality scores Genome Res 2008 18 11 1851 1858 10.1101/gr.078212.108 18714091 PMC2577856

[B46] Schnidelman G Morikami A Jung J Baskin TI Carpita NC Derbyshire P McCann MC Benfey PN COBRA encodes a putative GPI-anchored protein, which is polarly localized and necessary for oriented cell expansion in *Arabidopsis* Genes Dev 2001 15 9 1115 1127 10.1101/gad.879101 11331607 PMC312689

[B47] Langmead B Trapnell C Pop M Salzberg SL Ultrafast and memory-efficient alignment of short DNA sequences to the human gemone Genome Biol 2009 10 R25 10.1186/gb-2009-10-3-r25 19261174 PMC2690996

[B48] Nagalakshmi U Wang Z Waern K Shou C Raha D Gerstein M Snyder M The transcriptional landscape of the yeast genome defined by RNA sequencing Science 2008 320 5881 1344 1349 10.1126/science.1158441 18451266 PMC2951732

[B49] Ji W Zhou W Gregg K Yu N Davis S Davis S A method for cross-species gene expression analysis with high-density oligonucleotide arrays Nucl Acids Res 2004 32 e93 10.1093/nar/gnh084 15247326 PMC443552

[B50] Yang SS Valdés-López O Xu WW Bucciarelli B Gronwald JW Hernández G Vance CP Transcript profiling of common bean (*Phaseolus vulgaris *L.) using the GeneChip^® ^Soybean Genome Array: optimizing analysis by masking biased probes BMC Plant Biol 2010 10 85 10.1186/1471-2229-10-85 20459672 PMC3017814

[B51] Czechowski T Stitt M Altmann T Udvardi MK Scheible WR Genome-wide identification and testing of superior reference genes for transcript normalization in Arabidopsis Plant Physiol 2005 139 1 5 17 10.1104/pp.105.063743 16166256 PMC1203353

[B52] Wang L Feng Z Wang X Wang X Zhang X DEGseq: an R package for identifying differentially expressed genes from RNA-seq data Bioinformatics 2010 26 136 138 10.1093/bioinformatics/btp612 19855105

[B53] Sampedro J Cosgrove DJ The expansin superfamily Genome Biol 2005 6 242 10.1186/gb-2005-6-12-242 16356276 PMC1414085

[B54] Pelloux J Rustérucci C Mellerowicz EJ New insights into pectin methylesterase structure and function Trends Plant Sci 2007 12 267 277 10.1016/j.tplants.2007.04.001 17499007

[B55] Taylor NG Scheible WR Cutler S Somerville CR Turner SR The *irregular xylem3 *locus of *Arabidopsis *encodes a cellulose synthase required for secondary cell wall synthesis Plant Cell 1999 11 769 779 10330464 10.1105/tpc.11.5.769 PMC144224

[B56] Zhong R Morrison WH III Freshour GD Hahn MG Ye ZH Expression of a mutant form of cellulose synthase AtCesA7 causes dominant negative effect on cellulose biosynthesis Plant Physiol 2003 132 786 795 10.1104/pp.102.019331 12805608 PMC167018

[B57] Bosca S Barton CJ Taylor NG Ryden P Neumetzler L Pauly M Roberts K Seifert GJ Interactions between MUR10/*Ces*A7 dependent secondary cellulose biosynthesis and primary cell wall structure Plant Physiol 2006 142 1353 1363 10.1104/pp.106.087700 17041031 PMC1676063

[B58] Baucher M Bernard-Vailhé MA Chabbert B Besle JM Opsomer C Van Montagu M Botterman J Down-regulation of cinnamyl alcohol dehydrogenase in transgenic alfalfa (*Medicago sativa *L.) and the effect on lignin composition and digestibility Plant Mol Biol 1999 39 437 447 10.1023/A:1006182925584 10092173

[B59] Guo D Chen F Inoue K Blount JW Dixon RA Down-regulation of caffeic acid 3-O-methyltransferase and caffeoyl CoA 3-O-methyltransferase in transgenic alfalfa (*Medicago sativa *L.): impacts on lignin structure and implications for the biosynthesis of G and S lignin Plant Cell 2001 13 73 88 11158530 10.1105/tpc.13.1.73 PMC102215

[B60] Reddy MSS Chen F Shadle G Jackson L Aljoe H Dixon RA Targeted down-regulation of cytochrome P450 enzymes for forage quality improvement in alfalfa (*Medicago sativa *L.) Proc Natl Acad Sci USA 2005 102 16573 16578 10.1073/pnas.0505749102 16263933 PMC1283808

[B61] Wooley R Ruth M Glassner D Sheehan J Process design and costing of bioethanol technology: a tool for determining the status and direction of research and development Biotechnol Prog 1999 15 794 803 10.1021/bp990107u 10514249

[B62] Wooley R Ruth M Sheehan J Ibsen K Majdeski H Galvez A Lignocellulosic biomass to ethanol process design and economics utilizing co-current dilute acid prehydrolysis and enzymatic hydrolysis: current and futuristic scenarios 1999 National Renewable Energy Laboratory, Golden CO, NREL/TP-580-26157

[B63] Aden A Ruth M Ibsen K Jechura J Neeves K Sheehan J Wallace B Montague L Slayton A Lukas J Lignocellulosic biomass to ethanol process design and economics utilizing co-current dilute acid prehydrolysis and enzymatic hydrolysis for corn stover 2002 National Renewable Energy Laboratory, Golden CO, NREL/TP-510-32438

[B64] Yang B Wyman CE Pretreatment: the key to unlocking low cost cellulosic ethanol Biofuels Bioproducts & Biorefining 2008 2 26 40 10.1002/bbb.4921324176

[B65] Turner SR Somerville CR Collapsed xylem phenotype of *Arabidopsis *identifies mutants deficient in cellulose deposition in the secondary cell wall Plant Cell 1997 9 689 701 9165747 10.1105/tpc.9.5.689 PMC156949

[B66] Taylor NG Laurie S Turner SR Multiple cellulose synthase catalytic subunits are required for cellulose synthesis in *Arabidopsis* Plant Cell 2000 12 2529 2539 11148295 10.1105/tpc.12.12.2529 PMC102235

[B67] Sauer N Molecular physiology of higher plant sucrose transporters FEBS Lett 2007 581 2309 2317 10.1016/j.febslet.2007.03.048 17434165

[B68] Riesmeier JW Hirner B Frommer WB Potato sucrose transporter expression in minor veins indicates a role in phloem loading Plant Cell 1993 5 1591 1598 8312741 10.1105/tpc.5.11.1591 PMC160388

[B69] Truernit E Sauer N The promoter of the Arabidopsis thaliana SUC2 sucrose-H+ symporter gene directs expression of beta-glucuronidase to the phloem: evidence for phloem loading and unloading by SUC2 Planta 1995 196 564 570 7647685 10.1007/BF00203657

[B70] Stadler R Truernit E Gahrtz M Sauer N The AtSUC1 sucrose carrier may represent the osmotic driving force for anther dehiscence and pollen tube growth in Arabidopsis Plant J 1999 19 269 278 10.1046/j.1365-313X.1999.00527.x 10476074

[B71] Barth I Meyer S Sauer N PmSUC3: characterization of a SUT2/SUC3-type sucrose transporter from *Plantago major* Plant Cell 2003 15 1375 1385 10.1105/tpc.010967 12782730 PMC156373

[B72] Meyer S Lauterbach C Niedermeier M Barth I Sjolund RD Sauer N Wounding enhances expression of AtSUC3, a sucrose transporter from Arabidopsis sieve elements and sink tissues Plant Physiol 2004 134 684 693 10.1104/pp.103.033399 14739351 PMC344544

[B73] Haigler CH Singh B Wang G Zhang D Paterson AH Genomics of cotton fiber secondary wall deposition and cellulose biogenesis Genetics and Genomics of Cotton. Plant Genetics and Genomics: Crops and Models 3 2009 New York, USA: Springer Science Business Media 385 417

[B74] Somerville CR Cellulose synthesis in higher plants Annu Rev Cell Dev Biol 2006 22 53 78 10.1146/annurev.cellbio.22.022206.160206 16824006

[B75] Fujii S Hayashi T Mizuno K Sucrose synthase is an integral component of the cellulose synthesis machinery Plant Cell Physiol 2010 51 294 301 10.1093/pcp/pcp190 20056592

[B76] Rolland F Baena-Gonzalez E Sheen J Sugar sensing and signaling in plants: conserved and novel mechanisms Annu Rev Plant Biol 2006 57 675 709 10.1146/annurev.arplant.57.032905.105441 16669778

[B77] Poirier Y Thoma S Somerville C Schiefelbein J Mutant of *Arabidopsis *deficient in xylem loading of phosphate Plant Physiol 1991 97 1087 1093 10.1104/pp.97.3.1087 16668493 PMC1081126

[B78] Hamburger D Rezzonico E MacDonald-Comber Petétot J Somerville C Poirier Y Identification and characterization of the Arabidopsis PHO1 gene involved in phosphate loading to the xylem Plant Cell 2002 14 889 902 10.1105/tpc.000745 11971143 PMC150690

[B79] Stefanovic A Ribot C Rouached H Wang Y Chong J Belbahri L Delessert S Poirier Y Members of the PHO1 gene family show limited functional redundancy in phosphate transfer to the shoot, and are regulated by phosphate deficiency via distinct pathways Plant J 2007 50 982 994 10.1111/j.1365-313X.2007.03108.x 17461783

[B80] Ribot C Wang Y Poirier Y Expression analyses of three members of the AtPHO1 family reveal differential interactions between signaling pathways involved in phosphate deficiency and the responses to auxin, cytokinin, and abscisic acid Planta 2008 227 1025 1036 10.1007/s00425-007-0677-x 18094993

[B81] Chen YF Li LQ Xu Q Kong YH Wang H Wu WH The WRKY6 transcription factor modulates PHOSPHATE1 expression in response to low Pi stress in *Arabidopsis* Plant Cell 2009 21 3554 3566 10.1105/tpc.108.064980 19934380 PMC2798333

[B82] Teale WD Paponov IA Palme K Auxin in action: Signalling, transport and the control of plant growth and development Nat Rev Mol Cell Biol 2006 7 847 859 10.1038/nrm2020 16990790

[B83] Ye ZH Vascular tissue differentiation and pattern formation in plants Annu Rev Plant Biol 2002 53 183 202 10.1146/annurev.arplant.53.100301.135245 12221972

[B84] De Smet I Jürgens G Patterning the axis in plants - auxin in control Curr Opin Genet Dev 2007 17 337 343 10.1016/j.gde.2007.04.012 17627808

[B85] Lucas M Godin C Jay-Allemand C Laplaze L Auxin fluxes in the root apex co-regulate gravitropism and lateral root initiation J Exp Bot 2008 59 55 66 17720688 10.1093/jxb/erm171

[B86] Sundberg B Uggla C Tuominen H Savidge R, Barnett J, Napier R Cambial growth and auxin gradients Cell and Molecular Biology of Wood Formation 2000 Oxford, UK: BIOS Scientific Publishers 169 188

[B87] Nilsson J Karlberg A Antti H Lopez-Vernaza M Mellerowicz E Perrot-Rechenmann C Sandberg G Bhalerao RP Dissecting the molecular basis of the regulation of wood formation by auxin in hybrid aspen Plant Cell 2008 20 843 855 10.1105/tpc.107.055798 18424614 PMC2390731

[B88] Kim HJ Triplett BA Cotton fiber growth in planta and in vitro. Models for plant cell elongation and cell wall biogenesis Plant Physiol 2001 127 1361 1366 10.1104/pp.010724 11743074 PMC1540163

[B89] Bennett MJ Marchant A Green HG May ST Ward SP Millner PA Walker AR Schulz B Feldmann KA *Arabidopsis *AUX1 gene: a permease-like regulator of root gravitropism Science 1996 273 948 950 10.1126/science.273.5277.948 8688077

[B90] Zhao C Craig JC Petzold HE Dickerman AW Beers EP The xylem and phloem transcriptomes from secondary tissues of the *Arabidopsis *root-hypocotyl Plant Physiol 2005 138 2 803 818 10.1104/pp.105.060202 15923329 PMC1150398

[B91] Marchant A Bhalerao R Casimiro I Eklöf J Casero PJ Bennett M Sandberg G AUX1 promotes lateral root formation by facilitating indole-3-acetic acid distribution between sink and source tissues in the *Arabidopsis *seedling Plant Cell 2002 14 589 597 10.1105/tpc.010354 11910006 PMC150581

[B92] Kakani A Li G Peng Z Role of AUX1 in the control of organ identity during in vitro organogenesis and in mediating tissue-specific auxin and cytokinin interaction in *Arabidopsis* Planta 2009 229 645 657 10.1007/s00425-008-0846-6 19052775

[B93] Mattsson J Sung ZR Berleth T Responses of plant vascular systems to auxin transport inhibition Development 1999 126 2979 2991 10357941 10.1242/dev.126.13.2979

[B94] Klee H Estelle M Molecular genetic approaches to plant hormone biology Annu Rev Plant Physiol Plant Mol Biol 1991 42 529 551 10.1146/annurev.pp.42.060191.002525

[B95] Marchant A Kargul J May ST Muller P Delbarre A Perrot-Rechenmann C Bennett MJ AUX1 regulates root gravitropism in *Arabidopsis *by facilitating auxin uptake within root apical tissues EMBO J 1999 18 2066 2073 10.1093/emboj/18.8.2066 10205161 PMC1171291

[B96] Benková E Michniewicz M Sauer M Teichmann T Seifertová D Jürgens G Friml J Local, efflux-dependent auxin gradients as a common module for plant organ formation Cell 2003 115 591 602 10.1016/S0092-8674(03)00924-3 14651850

[B97] Blancaflor EB Masson PH Plant gravitropism. Unraveling the ups and downs of a complex process Plant Physiol 2003 133 1677 1690 10.1104/pp.103.032169 14681531 PMC1540347

[B98] Blilou I Xu J Wildwater M Willemsen V Paponov I Friml J Heidstra R Aida M Palme K Scheres B The PIN auxin efflux facilitator network controls growth and patterning in *Arabidopsis *roots Nature 2005 433 39 44 10.1038/nature03184 15635403

[B99] Li X Chapple C Understanding lignification: challenges beyond monolignol biosynthesis Plant Physiol 2010 154 449 452 10.1104/pp.110.162842 20921161 PMC2948979

[B100] Bonawitz ND Chapple C The genetics of lignin biosynthesis: connecting genotype to phenotype Ann Rev Genet 2010 44 337 363 10.1146/annurev-genet-102209-163508 20809799

[B101] Boija E Johansson G Interactions between model membranes and lignin-related compounds studied by immobilized liposome chromatography Biochim Biophys Acta 2006 1758 620 626 10.1016/j.bbamem.2006.04.007 16733046

[B102] Kaneda M Rensing KH Wong JCT Banno B Mansfield SD Samuels AL Tracking monolignols during wood development in lodgepole pine Plant Physiol 2008 147 1750 1760 10.1104/pp.108.121533 18550683 PMC2492623

[B103] Marinova K Pourcel L Weber B Schwarz M Barron D Routaboul JM Debeaujon I Klein M The *Arabidopsis *MATE transporter TT12 acts as a vacuolar flavonoid/H+ -antiporter active in proanthocyanidin-accumulating cells of the seed coat Plant Cell 2007 19 6 2023 2038 10.1105/tpc.106.046029 17601828 PMC1955721

[B104] Zhao J Dixon RA MATE transporters facilitate vacuolar uptake of epicatechin 3'-O-glucoside for proanthocyanidin biosynthesis in *Medicago truncatula *and *Arabidopsis* Plant Cell 2009 21 2323 2340 10.1105/tpc.109.067819 19684242 PMC2751950

[B105] Yazaki K ABC transporters involved in the transport of plant secondary metabolites FEBS Lett 2006 580 1183 1191 10.1016/j.febslet.2005.12.009 16364309

[B106] Rea PA Plant ATP-binding cassette transporters Annu Rev Plant Biol 2007 58 347 375 10.1146/annurev.arplant.57.032905.105406 17263663

[B107] Usadel B Nagel A Steinhauser D Gibon Y Bläsing OE Redestig H Sreenivasulu N Krall L Hannah MA Poree F Fernie AR Stitt M PageMan: An interactive ontology tool to generate, display, and annotate overview graphs for profiling experiments BMC Bioinformatics 2006 7 535 10.1186/1471-2105-7-535 17176458 PMC1766370

[B108] Eisen MB Spellman PT Brown PO Botstein D Cluster analysis and display of genome-wide expression patterns Proc Natl Acad Sci USA 1998 95 14863 14868 10.1073/pnas.95.25.14863 9843981 PMC24541

[B109] Schmittgen TD Livak KJ Analyzing real-time PCR data by the comparative C_T _method Nat Protoc 2008 3 6 1101 1108 10.1038/nprot.2008.73 18546601

