# Supplementary figures and images for: Using RNA-Seq for gene identification, polymorphism detection and transcript profiling in two alfalfa genotypes with divergent cell wall composition in stems
Source: BMC Genomics. 2011 Apr 19;12:199. doi: 10.1186/1471-2164-12-199 (PMC3112146; doi:10.1186/1471-2164-12-199)

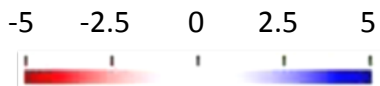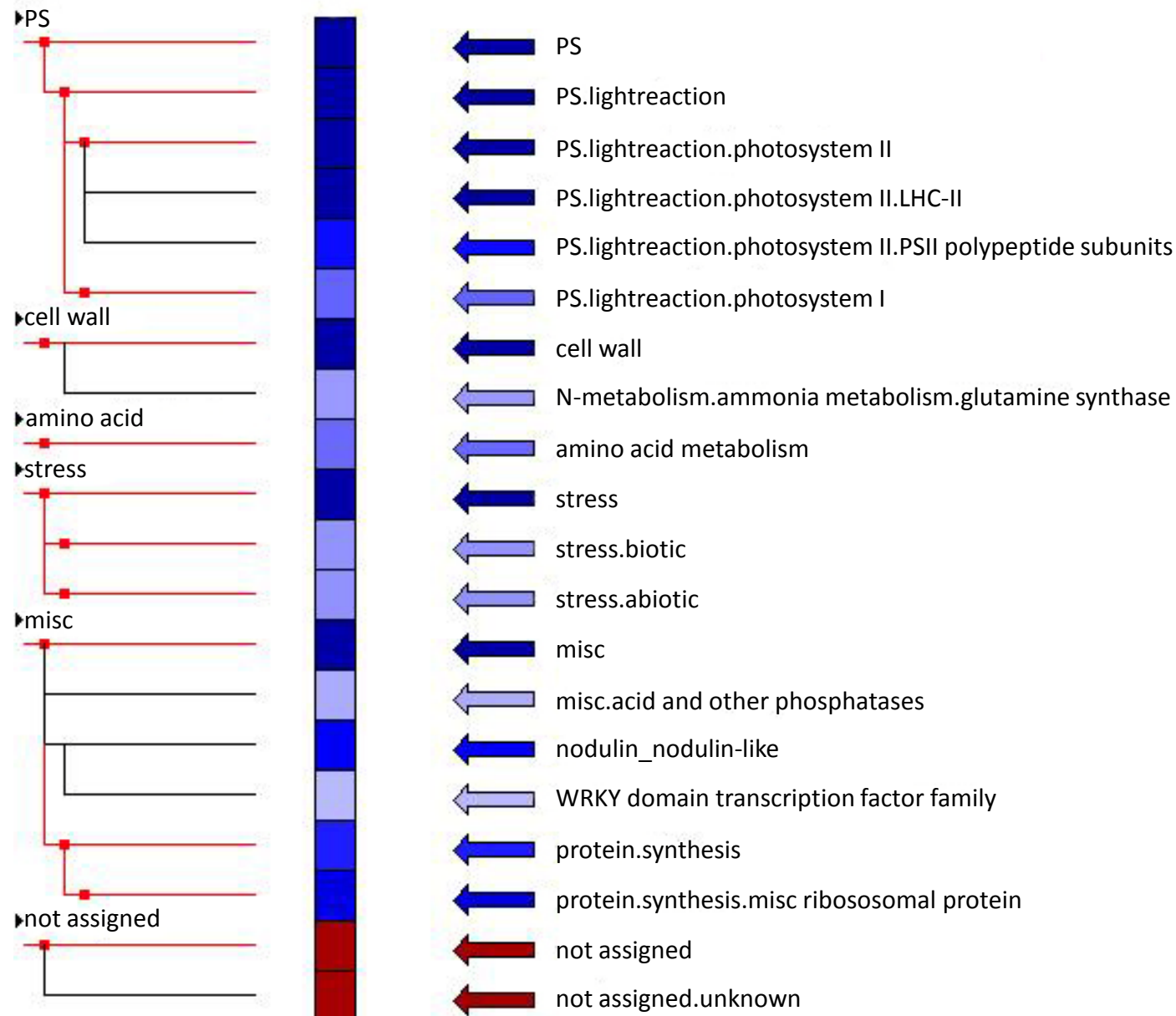

Supplement: Additional file 7 — Functional classes over- or under-represented among SNP-harboring genes. A figure showing the functional class over-representation analysis conducted for SNP-harboring genes. Functional classes that are over- or under-represented among SNP-harboring genes were identified using the PageMan over-representation analysis module. The z-vlaues for significant classes identified after Fisher's exact test with Bonferroni correction (z-value cutoff of 1) were false color coded using a scale of -5 to +5. The intensity of blue and red indicate the degree of over- and under-representation of the corresponding class, respectively. [file 1471-2164-12-199-S7.PDF]

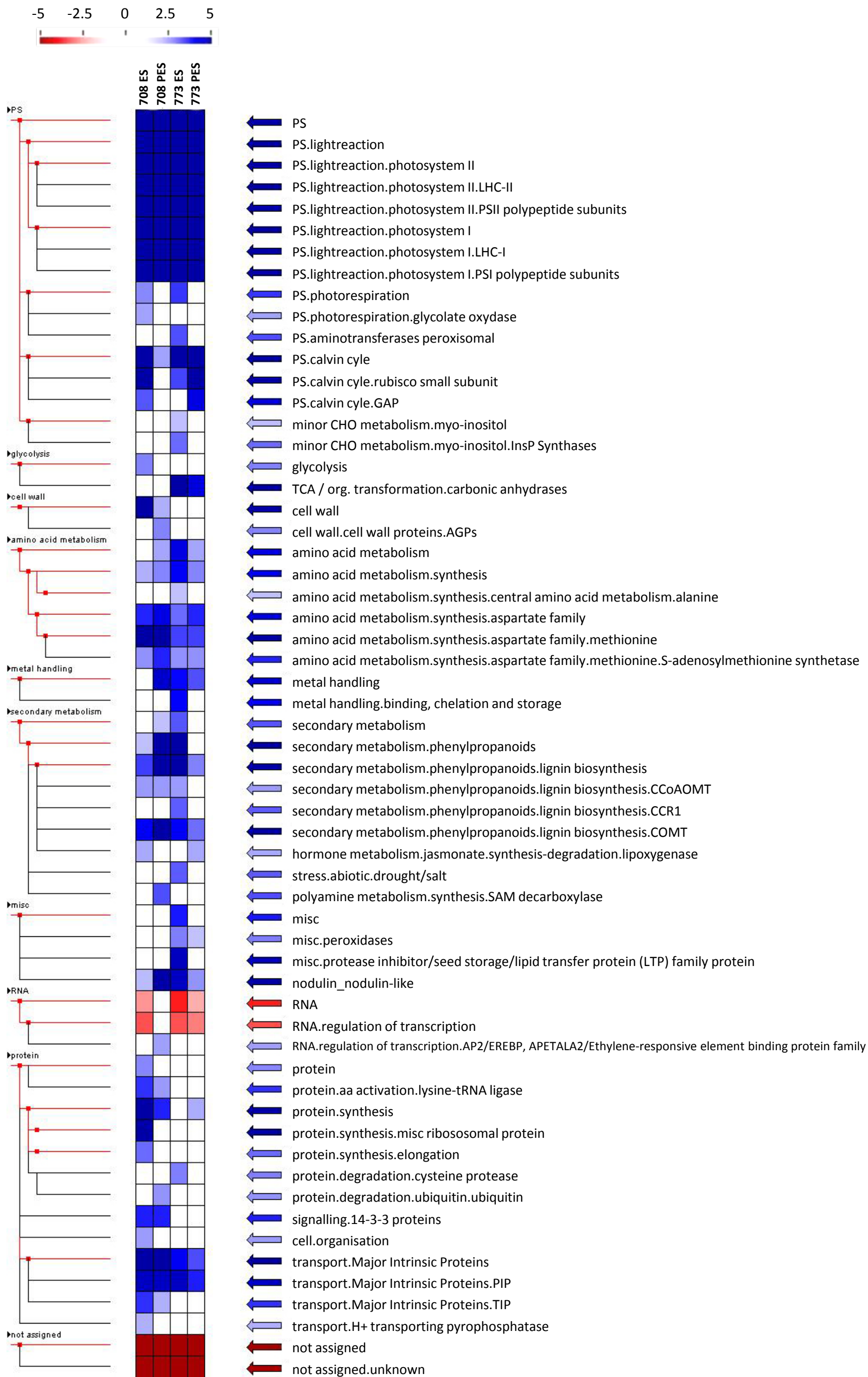

Supplement: Additional file 13 — Functional classes over- or under-represented among the top 500 most abundant transcripts in each library. A figure showing the results from functional class over-representation analysis for the top 500 most abundant transcripts in ES and PES internodes of alfalfa genotypes 708 and 773. For details, see the description for additional file 7. [file 1471-2164-12-199-S13.PDF]

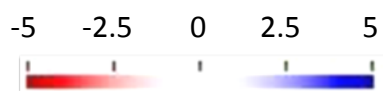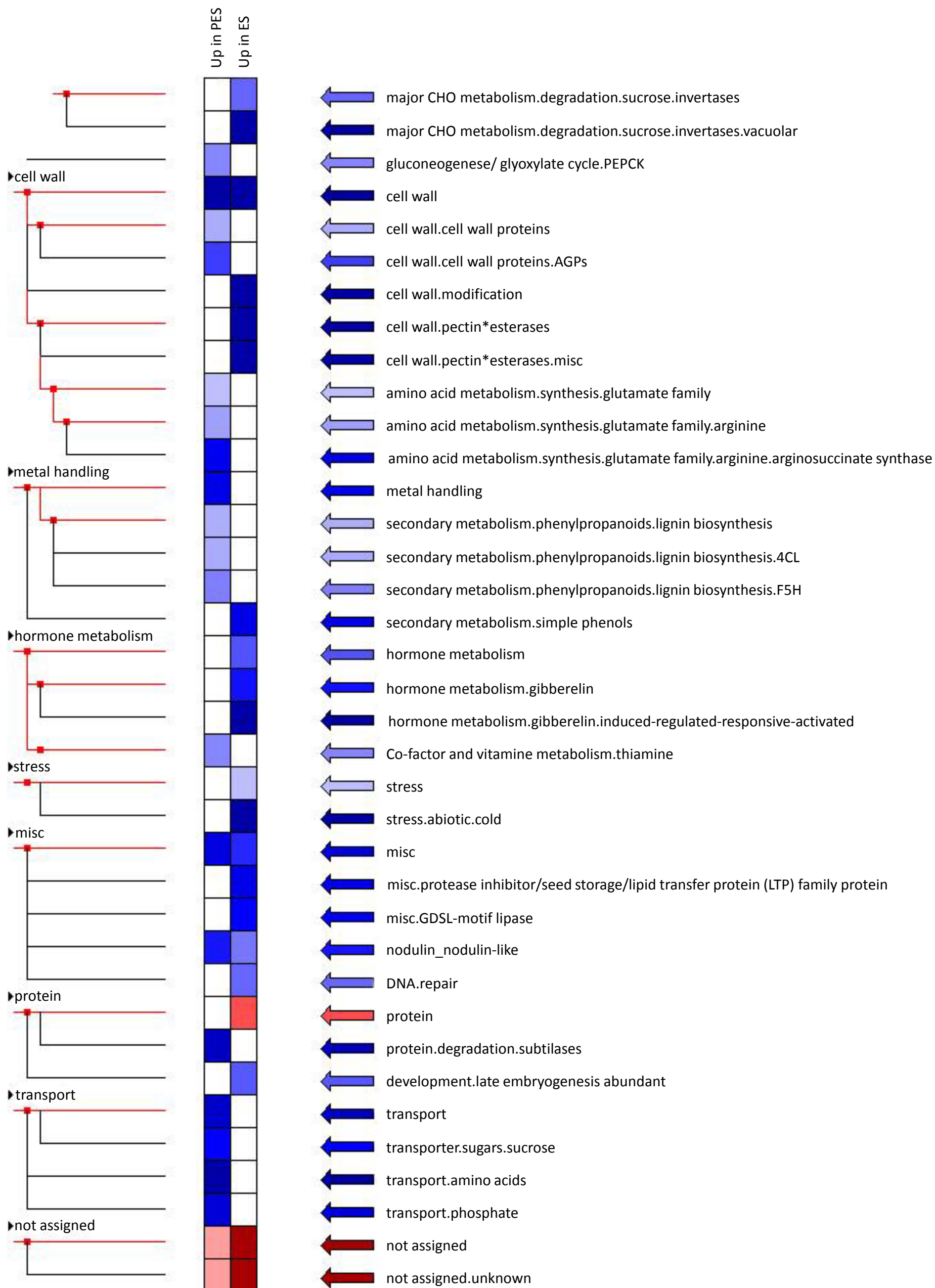

Supplement: Additional file 23 — Functional classes over- or under-represented among genes involved in general stem development independent of genotypic variation in alfalfa. A figure showing the functional class over-representation analysis for genes involved in general stem development independent of genotypic variation in alfalfa (Log2(PES/ES)≥1 or ≤-1 in both genotypes 708 and 773). "Up in PES" and "Up in ES" indicate genes up-regulated in PES and ES internodes in both genotypes, respectively. For details, see the description for additional file 7. [file 1471-2164-12-199-S23.PDF]

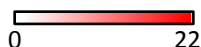

708 ES  
708 PES  
773 ES  
773 PES

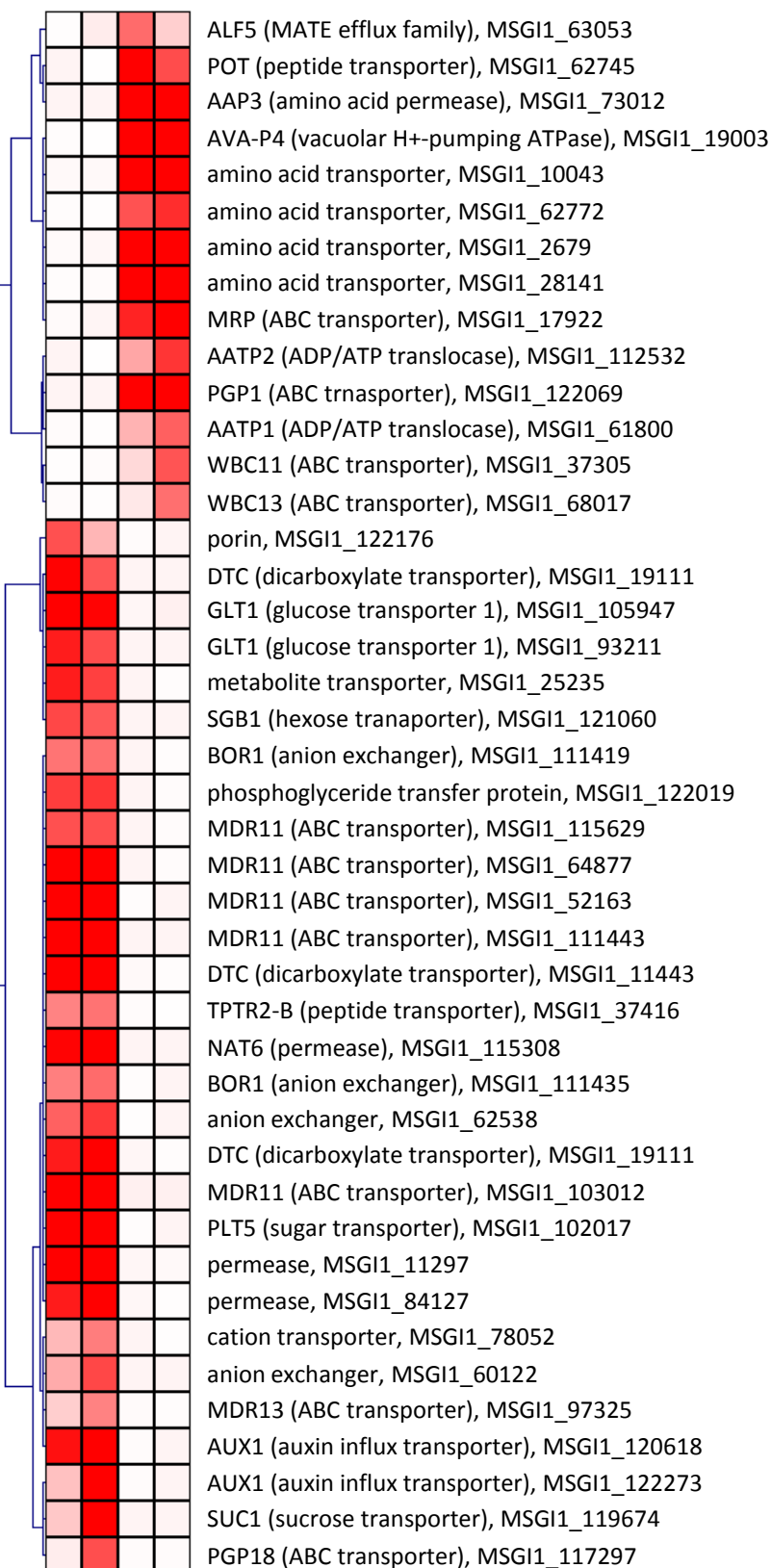

Supplement: Additional file 25 — Hierarchical clustering analysis of selected transporter genes differentially expressed between 708 and 773 in both ES and PES internodes. A figure showing a heatmap for 42 transporter genes differentially expressed between 708 and 773 in both ES and PES internodes (p < 0.001, FDR < 0.025, ≥ 2-fold difference). The RPKM-normalized expression counts for each gene in each library are represented by the intensity of the red color on a 0 to 22 scale. Dark red (scale intensity 22) indicates genes with RPKM-normalized expression counts ≥ 22. See Methods for details. A complete list of the transporter genes selected, RPKM-normalized expression counts, and corresponding MapMan functional categories are provided in Additional file 24. [file 1471-2164-12-199-S25.PDF]

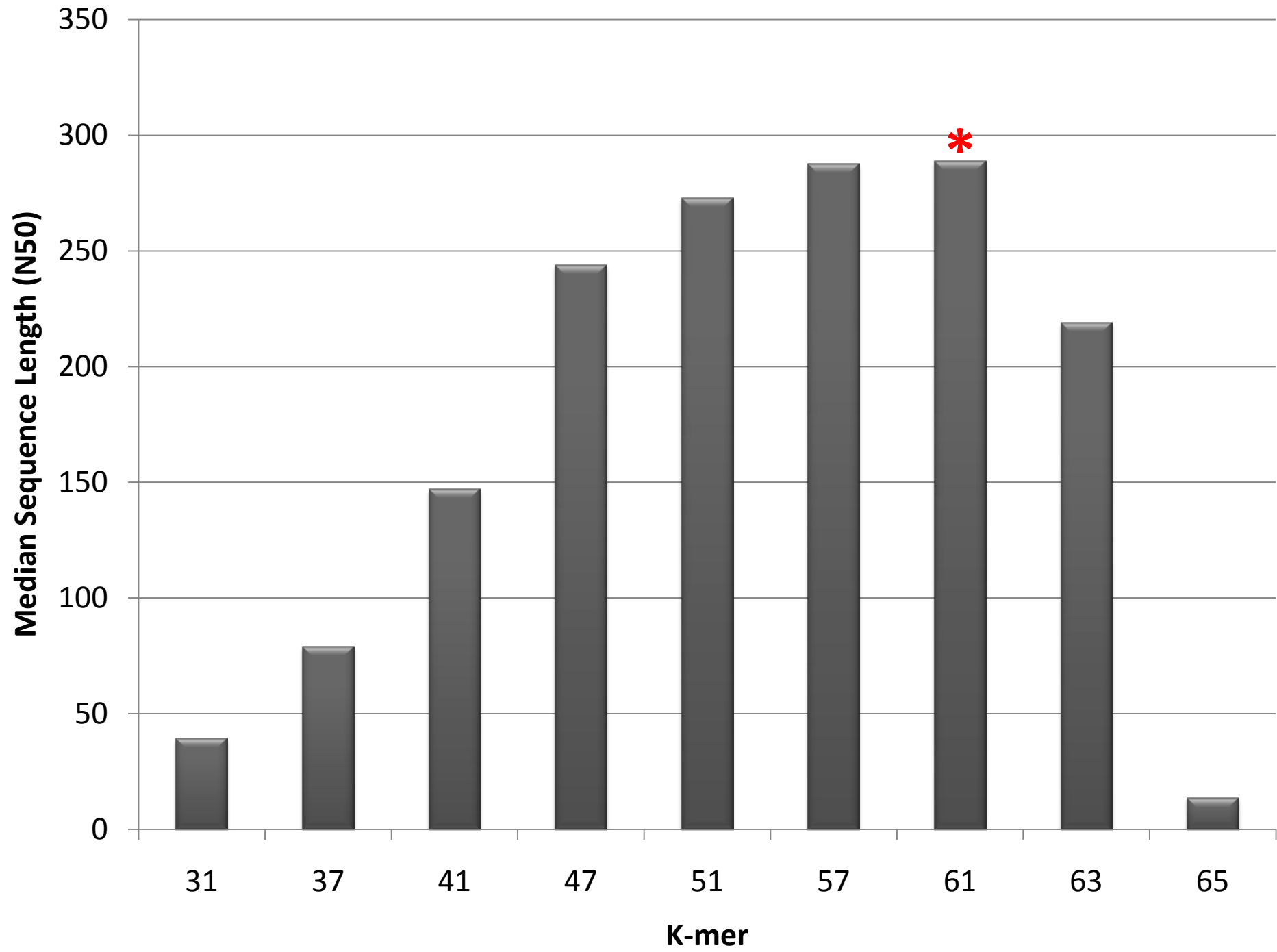

Supplement: Additional file 26 — Optimization of de novo assembly of Illumina GA-II EST reads with a series of k-mers using the Velvet program [32]. A figure showing the median sequence length of the contigs (y-axis) for a series of k-mers (31, 37, 41, 47, 51, 57, 61, 63, 65) tested using the Velvet program. k-mer 61 produced the longest median sequence length. [file 1471-2164-12-199-S26.PDF]
